# Antiviral Activity of Chlorophyll Extracts from *Tetraselmis* sp., a Marine Microalga, Against Zika Virus Infection

**DOI:** 10.3390/md22090397

**Published:** 2024-08-31

**Authors:** Nalae Kang, Eun-A Kim, Areumi Park, Seong-Yeong Heo, Jun-Ho Heo, Won-Kyu Lee, Yong-Kyun Ryu, Soo-Jin Heo

**Affiliations:** 1 Jeju Bio Research Center, Korea Institute of Ocean Science and Technology (KIOST), Jeju 63349, Republic of Korea; nalae1207@kiost.ac.kr (N.K.); euna0718@kiost.ac.kr (E.-A.K.); areumi1001@kiost.ac.kr (A.P.); syheo@kiost.ac.kr (S.-Y.H.); unknown0713@kiost.ac.kr (J.-H.H.); wonkyulee@kiost.ac.kr (W.-K.L.); ykyou0111@kiost.ac.kr (Y.-K.R.); 2 Department of Biology, University of Science and Technology (UST), Daejeon 34113, Republic of Korea

**Keywords:** *Tetraselmis* sp., microalga, chlorophyll, antiviral activity, zika virus

## Abstract

Recent advancements in the large-scale cultivation of *Tetraselmis* sp. in Korea have enabled year-round production of this marine microalgae. This study explores the potential industrial applications of *Tetraselmis* sp. biomass by investigating the antiviral properties of its extracts and primary components. The antiviral effects of *Tetraselmis* sp. extracts were evaluated in Zika virus (ZIKV)-infected cells. Following extensive isolation and purification, the main compounds were characterized using liquid chromatography–mass spectrometry (LC-MS) and nuclear magnetic resonance (NMR) analyses. Their antiviral activities were confirmed using in vitro and in silico tests. *Tetraselmis* sp. extracts reduced infectious viral particles and non-structural protein 1 messenger RNA levels in ZIKV-infected cells without inducing cytotoxicity. Additionally, they modulated the interferon-mediated immune system responses. *Tetraselmis* sp. extracts are composed of four main chlorophylls: chlorophyll a, chlorin e_6_-13^1^-15^2^-dimethyl-17^3^-phytyl ester, hydroxychlorophyll a, and hydroxypheophytin a. Among them, chlorophyll a, chlorin e_6_-13^1^-15^2^-dimethyl-17^3^-phytyl ester, and hydroxypheophytin showed the antiviral activities in ZIKV-infected cells and molecular docking simulations predicted interactions between these chlorophylls and ZIKV. Our findings suggest that *Tetraselmis* sp. chlorophyll extracts exert antiviral effects against ZIKV and could serve as potential therapeutic candidates against ZIKV infection.

## 1. Introduction

Microalgae, one of the oldest living organisms on Earth, are primary producers of autotrophic and mixotrophic microorganisms capable of photosynthesis [1,2]. Edible microalgae have been consumed as food for thousands of years, and dietary supplements containing microalgae have been considered safe for human use for several decades [3,4]. These organisms synthesize several bioactive compounds such as chlorophylls, carotenoids, proteins, and oils, which hold significant potential for the food and pharmaceutical industries [5]. Chlorophyll is one of the most abundant phytochemicals in microalgae and exhibits antioxidant [6,7,8], anti-inflammatory [9,10], anticancer [11,12], and anti-obesity properties [13].

Microalgae offer several advantages over macroorganisms for biotechnological applications, including shorter generation times, space-efficient cultivation in systems such as closed photobioreactors and open ponds, and adaptability to varying environmental conditions. Microalgae can adjust their biochemical composition depending on climate, growth phase, and cultivation conditions, such as light and inorganic carbon. Moreover, these organisms can utilize waste as nutrients and contribute to mitigating the greenhouse effect [14,15,16]. These attributes make microalgae a promising, sustainable, and cost-effective resource, spurring extensive research into large-scale production technologies [17,18,19].

Chlorophyta *Tetraselmis* sp. has been suggested to be a good source of active substances, such as pigments, lipids, and carotenoids [20,21,22]. In Korea, an innovative year-round cultivation system has been developed, using a semi-open raceway design within greenhouses to achieve large-scale production of *Tetraselmis* sp. [23,24]. The semi-open system was designed by installing open raceway ponds in a greenhouse to maximize biomass yield under varying seasonal conditions. Furthermore, *Tetraselmis* sp. ethanolic extracts have revealed antioxidant, antiviral, and anti-inflammatory properties, underscoring its potential for industrial applications [25]. However, the specific bioactivities of chlorophylls extracted from *Tetraselmis* sp. remain unexplored.

The Zika virus (ZIKV), a mosquito-borne, enveloped, positive-stranded RNA virus from the *Flaviviridae* family, continues to present a significant public health challenge, with no approved antiviral treatments available [26]. Several outbreaks have been reported across various regions, including Micronesia (2007); French Polynesia, New Caledonia, Easter Island, and Cook Island (2013–2014); Brazil, South America, Central America, North America, and the Caribbean (2015–2016); the Philippines, Vietnam, and Thailand (2015–2016); and Singapore (2016). By July 2019, the World Health Organization (WHO) reported autochthonous mosquito-borne transmission of ZIKV in 87 countries and territories across four of the six WHO regions (Africa, the Americas, Southeast Asia, and the Western Pacific) [26,27,28,29]. ZIKV infection is associated with several neurological complications, such as congenital microcephaly, Guillain-Barré syndrome, and meningoencephalitis [30]. The urgent necessity for effective antiviral agents against ZIKV represents a pressing scientific concern.

In this study, we investigated the antiviral properties of acetone extracts derived from *Tetraselmis* sp., a marine microalga, and characterized their primary bioactive components.

## 2. Results and Discussion

### 2.1. Inhibitory Effects of Tetraselmis *sp.* Acetone Extracts on ZIKV

**Figure 1:**
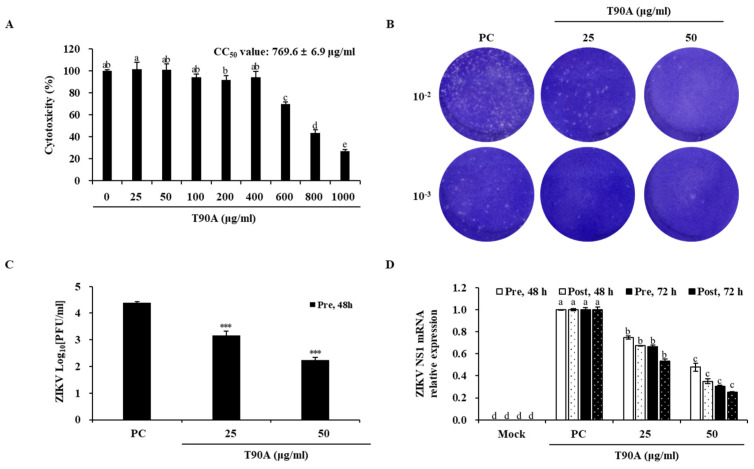
Inhibitory effects of T90A on ZIKV infection in Vero E6 cells. Cytotoxicity of T90A (**A**). Plaque assay image (**B**) and viral titer (**C**) illustrating the antiviral activity of T90A using plaque assay. Effect of T90A on ZIKV NS1 mRNA expression levels (**D**). Asterisks indicate significant differences at *** *p* < 0.001. The different lowercase letters indicate significant differences between each concentration for each experiment (Pre-48 h, Post-48 h, Pre-72 h, and Post-72 h) (one-way analysis of variance with post hoc Tukey’s test, *p* < 0.05).

Acetone extraction allows the obtention of secondary metabolites from plants, including chlorophyll [31,32,33,34]. *Tetraselmis* sp. extract, obtained using 90% acetone (T90A), yielded a green substance with an approximately 30% extraction yield. The inhibitory effects of T90A on ZIKV were tested in both Vero E6 cells and test tubes, a cell-free system. In Vero E6 cells, the inhibitory effect of T90A was assessed based on the viral plaque formation rate and in the expression levels of capsid non-structural protein 1 (NS1) messenger RNA (mRNA) (Figure 1). Non-infected and non-treated cells were defined as the mock group, and infected and non-treated cells were used as the positive control (PC) group. The cytotoxicity test revealed that T90A showed half-maximal cytotoxic concentrations (CC_50_) of 769.6 ± 6.9 μg/mL in Vero E6 cells (Figure 1A). Subsequent assays demonstrated that T90A, at concentrations of 25 and 50 µg/mL, significantly inhibited ZIKV replication, reducing viral titers by 20- and 218-fold, respectively, without cytotoxicity (Figure 1B,C). Infected cells exhibited a plaque-forming virus titer of 24,700 ± 1838 PFU/mL, whereas the T90A-treated cells exhibited reduced titers of 1200 ± 1039 at 25 µg/mL and 113 ± 103 PFU/mL at 50 µg/m (Figure S1). Additionally, T90A reduced the expression of the NS1 mRNA in a dose-dependent manner, with effective concentration (EC_50_) values of 36.4 ± 0.5 and 31.3 ± 0.8 µg/mL at 72 h pre- and post-infection, respectively, resulting in selectivity index (SI) values of 21.1 and 24.6 (Figure 1D and Table 1).

**Figure 2:**
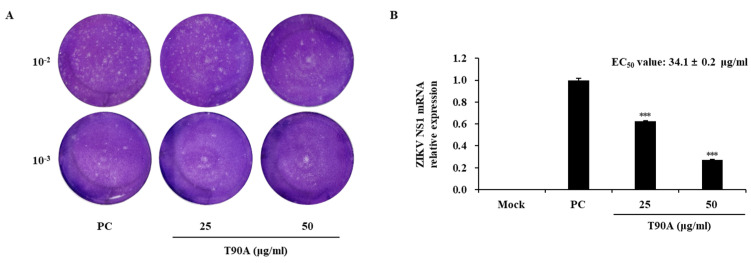
Virucidal effects of T90A on ZIKV. Plaque assay images (**A**) and NS1 mRNA expression levels (**B**) demonstrating the virucidal effect of T90A. Asterisks indicate significant differences at *** *p* < 0.001 (one-way analysis of variance with post hoc Tukey’s test, *p* < 0.05).

The virucidal effect of T90A on ZIKV was further confirmed by evaluating its impact on the viral plaque formation rate and NS1 mRNA levels. T90A promoted a concentration-dependent decrease in viral titer and NS1 mRNA levels, with an EC_50_ value of 34.1 ± 0.2 and SI value of 22.6 (Figure 2 and Table 1). Recent research has explored the antiviral properties of natural compounds. For example, Mulberry (*Morus* spp.) methanolic and aquatic extracts (50 µg/mL) exhibited an inhibitory rate of 34%–45% against the coronavirus [35]. Additionally, ethanolic extracts from *Tecoma* sp. leaves exhibited EC_50_ values of 66.79–131.0 µg/mL against ZIKV [36], while the essential oil from *Lippia alba* showed antiviral activity with EC_50_ values of 32.2 µg/mL (virucidal activity) and 54.1 µg/mL (post-treatment) [37].

In this study, T90A exhibited 31.3–36.4 µg/mL of antiviral activity and virucidal activity, underscoring its potential as an antiviral agent compared to other extracts from natural sources previously reported.

### 2.2. Effect of T90A on Interferon Response in ZIKV-Infected Cells

**Figure 3:**
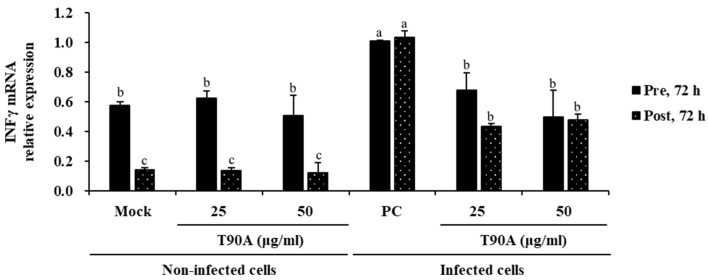
Impact of T90A on IFNγ levels in non-infected and ZIKV-infected cells. The different lowercase letters indicate significant differences between each tested cell for each experiment (Pre-72 h and Post-72 h) (one-way analysis of variance with post hoc Tukey’s test, *p* < 0.05).

Interferons (IFNs) are cytokines secreted by host cells during an antiviral immune response, inhibiting the translation of viral proteins and viral replication [38,39]. In particular, IFN gamma (IFNγ) inhibits viral invasion by regulating the expression and/or distribution of receptors required for virus entry and by impeding the transfer step of the invading virus from the endosome into the cytoplasm [40]. Additionally, IFN-γ exert an antagonistic effect on the production of immunosuppressive and anti-inflammatory interleukins [41]. The impact of T90A on IFNγ was assessed by measuring IFNγ mRNA levels in both non-infected and ZIKV-infected cells. In non-infected cells, T90A pre- and post-treatment did not significantly affect the expression of IFNγ. In contrast, in ZIKV-infected cells, the T90A treatment at 25 and 50 µg/mL significantly decreased IFNγ mRNA levels compared to that of the PC group (Figure 3). A previous study demonstrated that the widely used drugs minocycline and nimesulide inhibit the inflammatory response by reducing IFNγ production triggered by the SARS-CoV-2 Spike protein in Vero E6 cells [42]. Based on these facts, our findings suggest that T90A modulated the immune response in ZIKV-infected cells by reducing IFNγ expression without inducing an auto-stimulatory effect.

### 2.3. Isolation and Purification of Main Compounds from T90A

**Figure 4:**
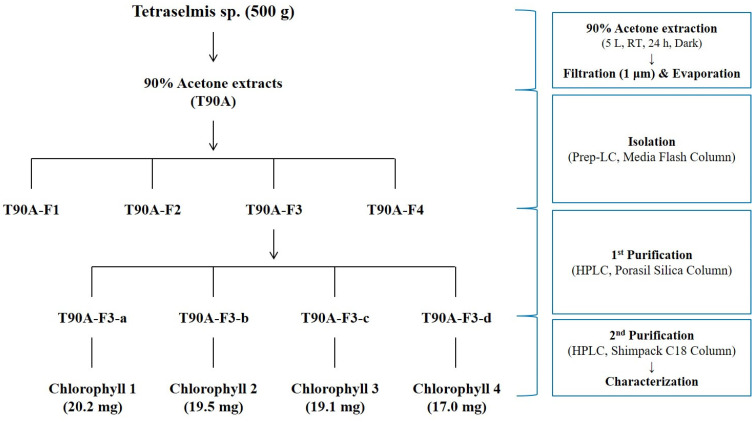
Isolation and purification scheme of main compounds from T90A.

**Figure 5:**
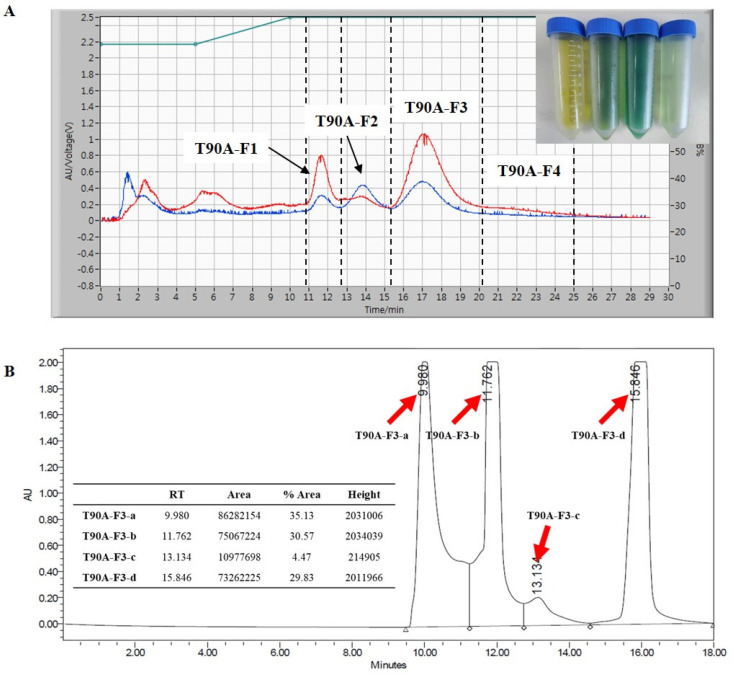
Isolation of T90A. Prep-LC chromatogram of T90A (**A**) and HPLC chromatogram of T90A-F3 (**B**).

**Figure 6:**
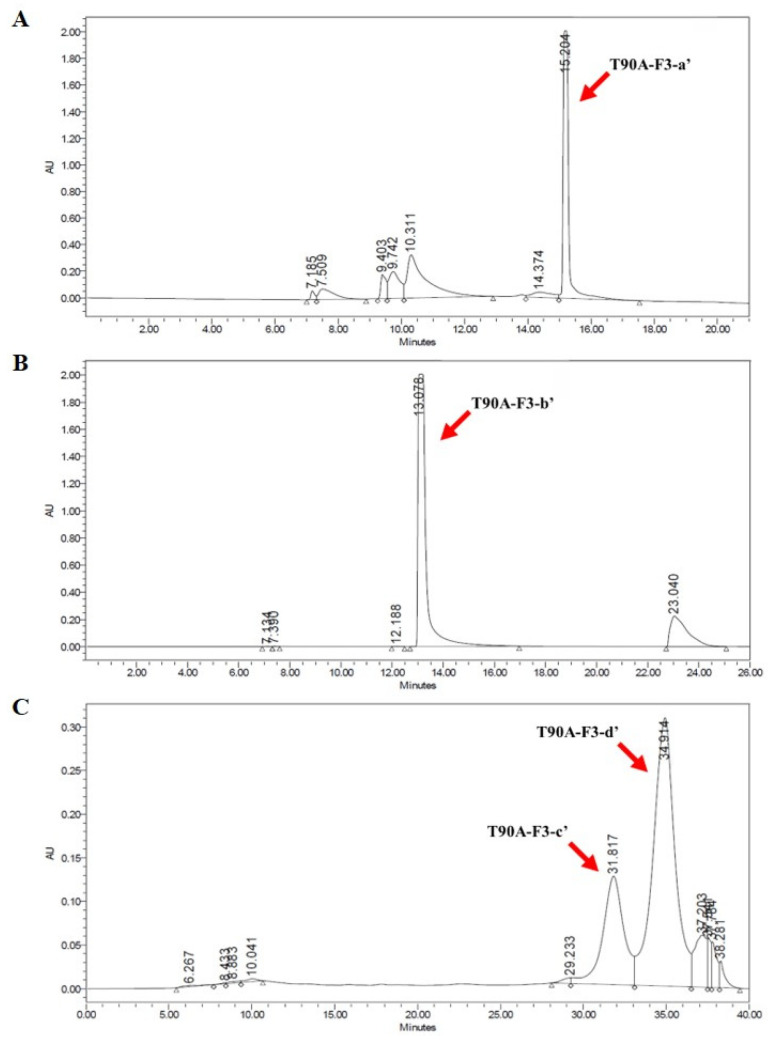
Purification of T90A-F3. HPLC chromatogram of T90A-F3-a’ (**A**), T90A-F3-b’ (**B**), and T90A-F3-c’ and T90A-F3-d’ (**C**).

The isolation and purification of main compounds from T90A involved several steps (Figure 4). Using preparative liquid chromatography (Prep-LC) with a Media Flash Column, four primary target fractions were isolated from T90A, detected at wavelengths of 254 nm (blue line) and 400 nm (red line). These fractions were designated as T90A-F1 (yellow), T90A-F2 (light green), T90A-F3 (green), and T90A-F4 (light green) (Figure 5A). Peak intensities of T90A-F1, T90A-F3, and T90A-F4 were the highest at 400 nm, whereas that of T90A-F2 was the highest at 254 nm. T90A-F3 showed the highest peaks at both wavelengths and was selected for further purification and analysis using a silica column (Porasil silica column). This step yielded another four fractions: T90A-F3-a, T90A-F3-b, T90A-F3-c, and T90A-F3-d (Figure 5B). Among these, the three main targets showed similar percentage area (% area) values in the chromatogram (T90A-F3-a: 35.13%, T90A-F3-b: 30.57%, and T90A-F3d: 29.83%); therefore, these fractions were subjected to the next step of purification using a C18 column (Shimpack C18 column). After purification of T90A-F3-a, seven peaks were identified, with the seventh fraction (denoted as T90A-F3-a’) representing 48.19% of the total area and 99.08% purity (Figure 6A and Table S1). For T90A-F3-b, five peaks were observed following purification, with the fourth fraction (denoted as T90A-F3-b’) exhibiting 80.99% area and 98.05% purity (Figure 6B and Table S2). For T90A-F3-d, seven peaks were obtained after purification, with the sixth (denoted as T90A-F3-c’; purity: 94.68%) and seventh (denoted as T90A-F3-d’; purity: 99.61%) fractions accounting for 24.25% and 61.88% of the area, respectively, showing high purities (Figure 6C and Table S3).

### 2.4. Characterization of Four Main Compounds from T90A

The structures of the purified compounds T90A-F3-a’, T90A-F3-b’, T90A-F3-c’, and T90A-F3-d’, were confirmed using LC–mass spectrometry (LC/MS), ^1^H, ^13^C, and two-dimensional (2D) nuclear magnetic resonance (NMR; Heteronuclear single quantum coherence spectroscopy [HSQC] and Heteronuclear multiple bond correlation [HMBC]) analyses. Detailed mass and NMR spectra are provided in the Supplementary Information (Figures S2–S21).

T90A-F3-a’ (chlorophyll a, C_55_H_72_O_5_N_4_Mg, 893 MW): positive electrospray ionization (ESI)-MS *m*/*z* 931 [M + K]^+^; ^1^H-NMR (600 MHz, acetone-d6, δH): 9.76 (1H, s, H-b), 9.43 (1H, s, H-a), 8.59 (1H, s, H-d), 8.12 (1H, dd, *J* = 18.0, 5.4 Hz, H-2a), 6.23 (1H, dd, *J* = 18.0, 1.2 Hz, H-2b-a), and 6.01 (1H, dd, *J* = 5.4, 1.2 Hz, H-2b-b); ^13^C-NMR (150 MHz, acetone-d6, δC): 192.2 (C-9), 173.2 (C-7c), 173.1 (C-10a), 169.1 (C-18), 161.5 (C-16), 157.5 (C-17), 156.8 (C-11), 154.7 (C-13), 147.9 (C-12), 147.6 (C-15), 146.1 (C-14), 146.0 (C-4), 143.9 (C-P3), 141.8 (C-2), 139.0 (C-1), 135.2 (C-5), 133.8 (C-3), 133.6 (C-6), 107.3 (C-c), 130.5 (C-2a), 119.3 (C-P2), 108.1 (C-b), 100.1 (C-a), 93.3 (C-d), 118.7 (C-2b), 60.5 (C-P1), 52.4 (C-10b), 60.6 (C-10), 51.2 (C-7), 48.8 (C-8), 32.5 (C-P11), 32.5 (C-P7), 27.8 (C-P15), 39.5 (C-P4), 39.4 (C-P14), 37.1 (C-P8), 37.1 (C-P10), 37.1 (C-P12), 36.3 (C-P6), 30.8 (C-7a), 30.4 (C-7b), 24.8 (C-P5), 24.8 (C-P13), 24.5 (C-P9), 17.1 (C-4a), 22.5 (C-8a), 22.1 (C-P16), 21.9 (C-P15a), 19.2 (C-P7a), 19.2 (C-P11a), 15.4 (C-4b), 11.9 (C-P3a), 11.8 (C-1a), 11.6 (C-5a), and 10.2 (C-3a).

**Figure 7:**
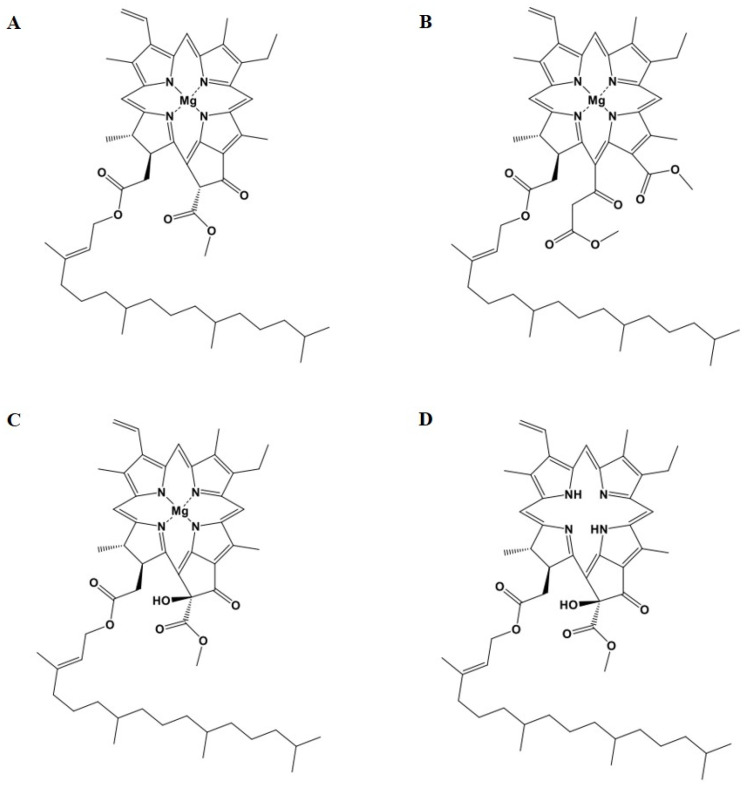
Structures of chlorophylls extracted from T90A. Chlorophyll a (**A**), chlorin e_6_-13^1^-15^2^-dimethyl-17^3^-phytyl ester (**B**), hydroxychlorophyll a (**C**), and hydroxypheophytin a (**D**).

The mass spectrum of T90A-F3-a’ showed *m*/*z* 931 [M + K], consistent with a potassium adduct chlorophyll a reported in a previous study [43]. The 1H-NMR spectrum of T90A-F3-a’ showed resonances for three olefin methane, three exo-methylene, and multiple methylene and methyl proton signals, indicating a phytol structure [44]. The 13C-NMR spectrum of T90A-F3-a’ showed resonances for 55 peaks, such as ketone (192.2 (C-9)), carboxyl (173.2 (C-7c), 173.1 (C-10a)), nitro olefin quaternary (169.1 (C-18), 161.5 (C-16), 157.5 (C-17), 156.8 (C-11), 154.7 (C-13), 147.9 (C-12), 147.6 (C-15), and 146.1 (C-14)), olefine quaternary (146.0 (C-4), 143.9 (C-P3), 141.8 (C-2), 139.0 (C-1), 135.2 (C-5), 133.8 (C-3), 133.6 (C-6), and 107.3 (C-c)), olefin methane (130.5 (C-2a), 119.3 (C-P2), 108.1 (C-b), 100.1 (C-a), and 93.3 (C-d)), olefin methylene (118.7 (C-2b)), oxygenated methylene (60.5 (C-P1)), methoxyl (52.4 (C-10b)), methine (60.6 (C-10), 51.2 (C-7), 48.8 (C-8), 32.5 (C-P11), 32.5 (C-P7), and 27.8 (C-P15)), methylene (39.5 (C-P4), 39.4 (C-P14), 37.1 (C-P8), 37.1 (C-P10), 37.1 (C-P12), 36.3 (C-P6), 30.8 (C-7a), 30.4 (C-7b), 24.8 (C-P5), 24.8 (C-P13), 24.5 (C-P9), and 17.1 (C-4a)), methyl (22.5 (C-8a), 22.1 (C-P16), 21.9 (C-P15a), 19.2 (C-P7a), 19.2 (C-P11a), 15.4 (C-4b), 11.9 (C-P3a), 11.8 (C-1a), 11.6 (C-5a), and 10.2 (C-3a)) carbon signals. The bond positions of each functional group were confirmed using HSQC and HMBC spectra. T90A-F3-a was identified as containing chlorophyll a (Figure 7A and Supplementary Figures S2–S6).

T90A-F3-b’ (chlorin e_6_-13^1^-15^2^-dimethyl-17^3^-phytyl ester, C_56_H_74_O_7_N_4_Mg, 938 MW): positive ESI-MS *m*/*z* 939 [M + H]^+^, 961 [M + Na]^+^. The mass spectrum of T90A-F3-b’ showed *m*/*z* 939 [M + H] and 961 [M + Na]. The 1H-NMR spectrum of T90A-F3-b’ was consistent with that of T90A-F3-a’. The 13C-NMR spectrum revealed one carboxyl carbon and one methoxyl carbon that were not present in T90A-F3-a’. The bond positions of these functional groups were confirmed using HSQC and HMBC spectra. T90A-F3-b’ was confirmed to contain chlorin e_6_-13^1^-15^2^-dimethyl-17^3^-phytyl ester (Figure 7B and Supplementary Figures S7–S11).

T90A-F3-c’ (hydroxychlorophyll a, C_55_H_72_O_6_N_4_Mg, 908 MW): positive ESI-MS *m*/*z* 909 [M + H]^+^, 910 [M + 2H]^+^. These results indicated the presence of two compounds with molecular weights of 908 and 886 Da, with the former being the predominant component. The 1H- and 13C-NMR spectra of T90A-F3-c’ were similar to those of T90A-F3-a’. Additionally, T90A-F3-c’ exhibited one additional oxygenated methine signal compared to T90A-F3-a’, suggesting the presence of an extra hydroxyl group (chlorophyll a). Furthermore, the bond positions of each functional group, including the hydroxyl group, were confirmed using HSQC and HMBC spectra. T90A-F3-c was identified as containing hydroxychlorophyll a (Figure 7C and Supplementary Figures S12–S16).

T90A-F3-d’ (hydroxypheophytin a): positive ESI-MS *m*/*z* 887 [M + H]^+^. These results indicate the presence of two compounds with molecular weights of 908 and 886 Da, with the latter being the major component. The 1H- and 13C-NMR spectra of T90A-F3-d’ showed a pattern similar to that of T90A-F3-c’. The bond positions of each functional group were confirmed using HSQC and HMBC spectra. T90A-F3-d’ was identified as containing hydroxypheophytin a, with Mg desorbed from T90A-F3-c’ (hydroxychlorophyll a) (Figure 7D and Supplementary Figures S17–S21). Mass and NMR spectra showed that T90A-F3-c’ and T90A-F3-d’ contained the same two compounds, which were produced due to the desorption of Mg.

### 2.5. Inhibitory Effects of Chlorophylls from T90A on ZIKV Infection

**Figure 8:**
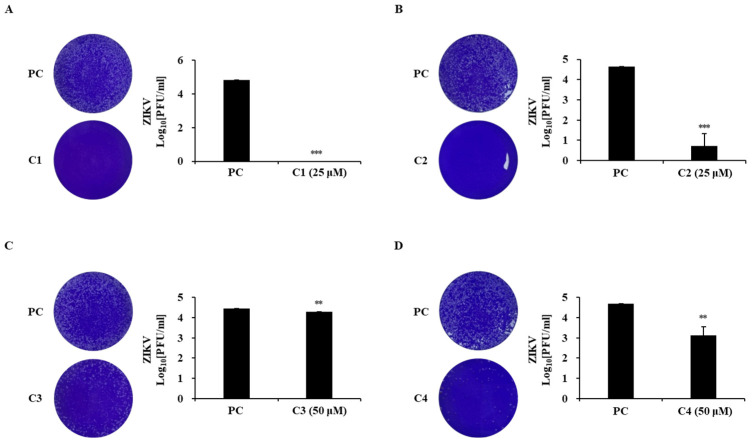
Reduction in infectious viral particles by chlorophylls during ZIKV infection. Plaque image and viral titers following treatment with chlorophyll a (**A**), chlorin e_6_-13^1^-15^2^-dimethyl-17^3^-phytyl ester (**B**), hydroxychlorophyll a (**C**), and hydroxypheophytin a (**D**). Asterisks indicate significant differences at ** *p* < 0.005, *** *p* < 0.001 (*t* tests, *p* < 0.05).

**Figure 9:**
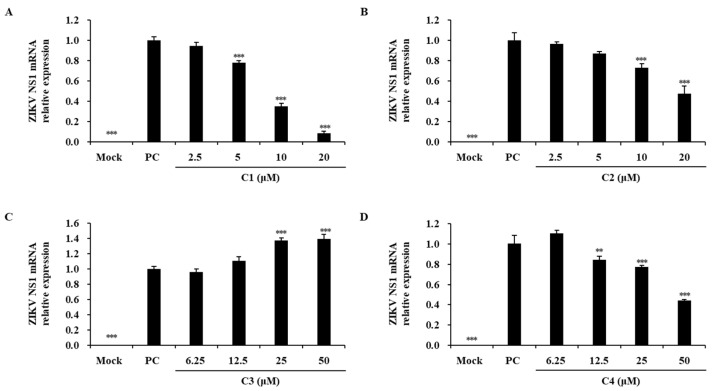
Effects of chlorophylls on ZIKV infection. NS1 mRNA expression levels in ZIKV-infected cells treated with chlorophyll a (**A**), chlorin e_6_-13^1^-15^2^-dimethyl-17^3^-phytyl ester (**B**), hydroxychlorophyll a (**C**), and hydroxypheophytin a (**D**). Asterisks indicate significant differences at ** *p* < 0.005, *** *p* < 0.001 (one-way analysis of variance with post hoc Tukey’s test, *p* < 0.05).

The antiviral properties of the isolated chlorophylls on ZIKV infection were assessed using the viral plaque formation rates and NS1 mRNA levels (Figure 8 and Figure 9). Chlorophyll a and chlorin e_6_-13^1^-15^2^-dimethyl-17^3^-phytyl ester at 25 μM reduced the viral particles by approximately 50,000-fold. These compounds also reduced NS1 mRNA levels in ZIKV-infected cells in a concentration-dependent manner across the 2.5–20 μM range (Figure 8A,B and Figure 9A,B). Hydroxychlorophyll a did not exhibit a significant antiviral effect, while hydroxypheophytin a at 50 μM reduced viral particles by approximately 130-fold and decreased NS1 mRNA levels in a concentration-dependent manner (12.5–50 μM) (Figure 8C,D and Figure 9C,D).

Previous studies have reported the antiviral activity of chlorophyll derivatives against various viruses, such as pheophorbide a against RNA viruses, including SARS-CoV-2 and West Nile virus [45,46,47], and chlorophyll c2 against infectious hematopoietic necrosis virus [48]; however, the antiviral activity of chlorophylls against ZIKV has not yet been investigated. Our findings provide new evidence for the antiviral potential of chlorophyll derivatives against ZIKV.

### 2.6. Molecular Docking Analysis of Chlorophylls to ZIKV Proteins

Depending on the ZIKV life cycle, the viral entry (envelope protein), polyprotein processing (NS2B/NS3), and viral replication (RNA-dependent RNA polymerase, RdRp) are regarded as potential therapeutic targets [49]. Although these proteins act within the host cells, since much research has been published on the absorption of chlorophylls and their derivatives by cells [11,50], these target proteins were applied for a molecular docking test to predict the mode-of-action of T90A (Table 2 and Table 3 and Supplementary Figures S22–S24). Chlorophyll a showed strong binding affinities with these proteins, with binding energies of −276.459, −767.837, and −692.708 kcal/mol, respectively. Chlorin e_6_-13^1^-15^2^-dimethyl-17^3^-phytyl ester also interacted with these proteins, with binding energies of −374.144, −779.602, and −727.470 kcal/mol, respectively (Table 2). These energy values indicate that these two chlorophylls from T90A exert a stronger impact on NS2B/NS3 and RdRp than on the envelope protein. Also, these chlorophylls formed several non-bond interactions with amino acids of the active site of each protein (Table 3). Notably, Mg^2+^ plays an essential role in stabilizing these interactions by forming complexes with both functional groups of chlorophylls and/or amino acids of the target proteins. Although hydroxychlorophyll a interacted with these proteins with binding energies of −511.311, −751.503, and −899.910 kcal/mol, respectively, Mg^2+^ of hydroxychlorophyll a interacts with the non-main amino acids of RdRp. This result implies that Mg^2+^ of hydroxychlorophyll a may have a negative impact on its antiviral efficacy (Tables S4 abd S5). Hydroxypheophytin a interacted with these proteins with relatively low binding energies of −122.571, −286.441, and −337.940 kcal/mol, respectively. However, the NH group of hydroxypheophytin a appeared to play an important role in forming bonds with amino acids of NS2B/NS3 and RdRp proteins (Tables S4 and S5).

## 3. Materials and Methods

### 3.1. Mass Production of Tetraselmis *sp.*

*Tetraselmis* sp. MBEyh04Gc (KCTC 12432BP), a marine microalga, was provided by the Marine Bioenergy R&D Consortium of Inha University, Incheon, Korea. The alga was cultured and mass-produced in a semi-open raceway system that allowed year-round cultivation, as described previously [24]. The harvested samples were lyophilized and stored at −80 °C until use.

### 3.2. Materials and Reagents

All solvents used for sample preparation were of analytical grade. LC-grade solvents were purchased from J.T. Baker (Clare, MI, USA). For the cell-based experiment, Dulbecco’s Phosphate-Buffered Saline (DPBS), Dulbecco’s Modified Eagle Medium (DMEM), DMEM/F12 powder, 100× L-glutamine, and Fetal Bovine Serum (FBS) were purchased from Gibco (Carlsbad, CA, USA). Penicillin–streptomycin was acquired from Sigma-Aldrich (St. Louis, MO, USA). ZIKV was provided by the Korea Centers for Disease Control and Prevention, and virus stocks of 1 × 10^5^ virus/mL were titrated and stored at −80 °C. TRIzol and diethylpyrocarbonate–water were purchased from Ambion Invitrogen (Waltham, MT, USA). Chloroform, isopropanol, and ethanol were purchased from EMSURE Merck & Co. (Rahway, NJ, USA). High-Capacity RNA-to-cDNA kit and Power SYBR Green PCR Master Mix were acquired from Applied Biosystems (Waltham, MT, USA).

### 3.3. Preparation of Extracts and Purification of Main Compounds

Lyophilized *Tetraselmis* sp. powder (500 g) was extracted with 90% acetone (5 L) at 21–25 °C in the dark for 24 h. The extracts were filtrated with 1 μm filter paper, evaporated under vacuum, and stored at −20 °C.

Three purification steps were performed to isolate and purify the main compounds from T90A. The crude extract was suspended in methanol and purified using a Prep-LC system with a Media Flash Column C18 (20 μm, 330 g) from Agela Technologies (Torrance, CA, USA). The column was eluted in gradient mode with a mobile phase solvent system containing water–methanol (0–5 min: 10:90, *v*/*v*; 5–10 min: 10:90 → 0:100, *v*/*v*; 10–30 min: 0:100, *v*/*v*) with a flow rate of 25 mL/min, with absorbance monitored at 254 and 400 nm. A second purification analysis was conducted using a Waters Delta 600 pump and Waters 486 tunable absorbance detector system (Framingham, MA, USA) with a Porasil silica column (5 μm, 20 mm × 250 mm). The column was eluted with a mobile phase solvent system containing hexane/ethyl acetate at a flow rate of 18 mL/min. A third purification analysis was performed using Waters Delta 600 and Waters 486 system (Framingham, MA, USA) with Shimpack C18 (5 μm, 20 mm × 250 mm). The column was eluted with a mobile phase solvent system containing water–methanol.

### 3.4. Structural Identification of Compounds

LC/MS was performed using a Waters Acuity H Class UPLC QDA (Framingham, MA, USA) with a Kinetex C18 (2.6 μm, 2.1 mm × 100 mm), scanning in the range of *m*/*z* 100–1050. One-dimensional and two-dimensional NMR experiments were conducted using an ASCEND 600 spectrometer (Bruker BioSpin GmbH, Rheinstetten, Germany).

### 3.5. Cell Culture

Vero E6 cells were purchased from the American Type Culture Collection (Washington, DC, NW, USA) and cultured and maintained in DMEM supplemented with 10% FBS and 1% penicillin–streptomycin at 37 °C in a 5% carbon dioxide (CO_2_) incubator.

### 3.6. Measurement of Cytotoxicity and Calculation of SI

Cytotoxicity was assessed using the 3-(4,5-Dimethyl-2-thiazolyl)-2,5-diphenyltetrazolium Bromide (MTT) assay [25]. Briefly, Vero E6 cells (1.4 × 10^4^ cells/well) were seeded in 96-well plates and incubated for 16 h. Cells were then treated with various concentrations of the samples and incubated for an additional 24 h. The SI was calculated as the ratio of the CC_50_ to the EC_50_.

### 3.7. Measurement of Viral Plaque Formation Rate

Vero E6 cells (1 × 10^6^ cells/well) were seeded in a 6-well plate and incubated until the formation of a monolayer. Cells were then washed with PBS, and 2 mL of DMEM/2% FBS was added. Samples were prepared for analysis by solubilizing in DMEM/2% FBS. For the sample pre-treatment experiment, the samples were pre-treated for 2 h before infection with ZIKV at a multiplicity of infection (MOI) of 0.01. For the sample post-treatment experiment, the samples were post-treated 2 h after infection with ZIKV at a MOI of 0.01. For the mock and PC groups, DMEM/2% FBS without sample or virus was used instead of samples and virus. After 48 h or 72 h of incubation, cell media were collected for plaque assay to titrate the number of viral particles.

The plaque assay was performed as previously described [51]. The cell culture medium was serially diluted by 10-fold with serum-free DMEM. Vero E6 cells (1 × 106 cells/well) were seeded in a 6-well plate and incubated until the formation of a monolayer. Cells were then washed twice with DPBS, and 0.2 mL of serum-free DMEM and 0.5 mL of each diluted cell culture media were added to the wall of the well. The cells were incubated for 2 h with gentle shaking every 15 min to facilitate virus adsorption. After adsorption, the inoculum was removed from the cells, and 3 mL of DMEM/F12-2% oxoid agarose was added to the cells. Plates were incubated at 37 °C in a 5% CO_2_ incubator for 5 days. Cells were then fixed with 4% formaldehyde for 1 h and stained with 0.1% crystal violet for 30 min. Viral titers were calculated using the following formula:

PFU/mL = Number of plaques/(dilution factor × volume of diluted virus/well)

### 3.8. Measurement of mRNA Expression Levels

Cells were cultured as described in Section 3.6, after which, they were assessed for the mRNA levels of ZIKV and monkey proteins. After incubation for 48 h or 72 h, the cells were used for the quantitative polymerase chain reaction (qPCR) assay to assess the mRNA levels of ZIKV and monkey proteins.

Total RNA was extracted using the acid guanidinium thiocyanate–phenol–chloroform extraction method [52], followed by the complementary DNA (cDNA) synthesis using a High-Capacity RNA-to-cDNA kit according to the manufacturer’s instructions. qPCR was performed using a Power SYBR Green PCR Master Mix (Applied Biosystems, Waltham, MT, USA) on a QuantStudio 3 real-time PCR system (Thermo Fisher Scientific, Waltham, MT, USA) under the following conditions: 95 °C for 10 min, followed by 40 cycles at 95 °C for 15 s, and 60 °C for 1 min. Expression levels were determined using the crossing point (Cp) method. All primers used in the present study have been previously described [53] and are listed in Table 4.

### 3.9. Measurement of Virucidal Effect

The virucidal assay was conducted in two steps, virucidal reactions and subsequent determination of viral infectivity, as previously described [54]. For the virucidal reactions, the sample was diluted 2× in DMEM, while the virus stock was diluted 1 × 10^5^ PFU/mL in DMEM. Equal volumes of the diluted sample and virus stock were mixed in a 1:1 ratio and incubated at 37 °C in a 5% CO_2_ incubator for 1 h. Following incubation, the viral infectivity of the mixture was determined using both plaque assay and qPCR assay, as described above in Section 3.7 and Section 3.8.

### 3.10. Molecular Docking Analysis between Chlorophylls and ZIKV Proteins

For molecular docking analysis, the three-dimensional (3D) structures of chlorophylls and ZIKV proteins were prepared using Discovery Studio 2024 (Biovia, San Diego, CA, USA). The 2D structures of chlorophylls derived from T90A were obtained and edited from PubChem (chlorophyll a, CID 12085802; hydroxychlorophyll a, CID 46174054; chlorin e_6_-13^1^-15^2^-dimethyl-17^3^-phytyl ester, modified form CID 12085802; hydroxypheophytin a, modified form CID46174054), and the 3D structures were optimized using a ligand preparation tool. The 3D structures of ZIKV proteins were obtained from the Protein Data Bank (envelope protein [5JHM], NS2B/NS3 [5LC0], and RdRp [5TFR]) and optimized using a protein preparation tool. Molecular docking analysis was conducted as previously described [55,56] using the CDOCKER protocol to calculate the binding energy and assess potential interactions.

### 3.11. Statistical Analysis

All experimental data are presented as the mean ± standard deviation (SD) from three independent experiments. Statistical analyses were conducted using one-way analysis of variance, followed by Tukey’s multiple comparison test and t tests, using GraphPad Prism software (version 9; GraphPad Software, San Diego, CA, USA). Statistical significance was set at *p* < 0.05 and 0.001.

## 4. Conclusions

The ability to mass-produce bioactive compounds is vital for industrial applications, and microalgae like *Tetraselmis* sp. offer a significant advantage due to their scalability and efficiency in production. In this study, we demonstrated the antiviral properties of *Tetraselmis* sp. extracts and their chlorophyll derivatives against ZIKV. Our results confirmed the feasibility of *Tetraselmis* sp. biomass in industrial applications. The main chlorophylls compounds—including chlorophyll a, chlorin e_6_-13^1^-15^2^-dimethyl-17^3^-phytyl ester, and hydroxypheophytin a—not only exhibit antiviral activity in ZIKV-infected cells but also show strong binding affinities to ZIKV proteins through molecular docking analysis. These findings suggest that *Tetraselmis* sp. chlorophyll extracts are potential candidates for future antiviral therapy against ZIKV. 

## Figures and Tables

**Table 1:** Antiviral activity of T90A on ZIKV-infected Vero E6 cells.

Antiviral Activity				Virucidal activity		Cytotoxicity
Pre-Treatment		Post-Treatment				
EC_50_ (μg/mL)	SI	EC_50_ (μg/mL)	SI	EC_50_ (μg/mL)	SI	CC_50_ (μg/mL)
36.4 ± 0.5	21.1	31.3 ± 0.8	24.6	34.1 ± 0.2	22.6	769.6 ± 6.9

EC_50_ and SI values for antiviral activity were calculated from the percentage of ZIKV NS1 mRNA inhibition in the cells treated with T90A for 72 h.

**Table 2:** Binding energies between chlorophylls and ZIKV proteins.

Chlorophyll	Protein	Binding energy (kcal/mol)
Chlorophyll a	Envelope protein	−276.459
	NS2B/NS3	−767.837
	RdRp	−692.708
Chlorin e_6_-13^1^-15^2^-dimethyl-17^3^-phytyl ester	Envelope protein	−374.144
	NS2B/NS3	−779.602
	RdRp	−727.470

**Table 3:** The non-bond interactions between chlorophylls and Zika virus proteins.

Chlorophyll	Protein	Non-Bond Interaction in Active Site
Chlorophyll a (C1)	Envelope protein	Mg^2+^-TRP101, Mg^2+^-GLY102, C1-LEU107 (2), C1-TRP101 (2)
	NS2B/NS3	Mg^2+^-ASN1152, Mg^2+^- GLY1153, Mg^2+^-VAL1154, Mg^2+^-VAL1162, C1-VAL1036, C1-HIS1051 (3), C1-ALA1132 (3), C1-SER1135 (2), C1-TYR1150, C1-GLY1151, C1-ASN1152 (2), C1-TYR1161 (4)
	RdRp	Mg^2+^-GLU707 (2), Mg^2+^-TRP848, C1-VAL708, C1-HIS713, C1-HIS732 (2), C1-CYS849,
Chlorin e_6_-13^1^-15^2^-dimethyl-17^3^-phytyl ester (C2)	Envelope protein	Mg^2+^-GLY104, Mg^2+^-Cys105, Mg^2+^-GLY106 C2-TRP101(5), C2-GLY104, C2-GLY106, C2-LEU107
	NS2B/NS3	Mg^2+^-ASP1129 (2), Mg^2+^-ASN1152, Mg^2+^-GLY1153, Mg^2+^-VAL1162, C2-HIS1051, C2-ALA1132, C2-VAL1154, C2-TYR1161 (2)
	RdRp	Mg^2+^-GLU707, C2-TRP702 (2), C2-VAL708 (2), C2-TRP848 (2)

The number in parentheses indicates the number of the non-bond interactions.

**Table 4:** Primer information.

Gene	Sequence	Primer
ZIKV NS1	5’-CRA CTA CTG CAA GYG GAA GG-3’	F
	5’-GCC TTA TCT CCA TTC CAT ACC-3’	R
Monkey GAPDH	5’-GCA AAT TCC ATG GCA CCG T-3’	F
	5’-TCG CCC CAC TTG ATT TTG G-3’	R
Monkey IFNγ	5’-CGA ATG TCC AAC GCA AAG CAG TAC-3’	F
	5’-TGC TCT TCG ACC TCG AAA CAT CTG-3’	R
Monkey IFIT1	5’- GGA TTC TGT ACA ATA CAC TAG AAA CCA-3’	F
	5’- CTT TTG GTT ACT TTT CCC CTA TCC-3 ‘	R
Monkey IFIT2	5’- ATC CCC CAT CGC TTA TCT CT-3’	F
	5’- CCACCTCAATTAATCAGGCACT-3’	R

## Data Availability

The original contributions presented in the study are included in the article/supplementary material, further inquiries can be directed to the corresponding author.

## Supplementary Materials

The following supporting information can be downloaded at: https://www.mdpi.com/article/10.3390/md22090397/s1. Figure S1: Inhibition effects of T90A on ZIKV infection; Figures S2–S6: Mass and NMR spectra of T90A-F3-a’; Figures S7–S11: Mass and NMR spectra of T90A-F3-b’; Figures S12–S16: Mass and NMR spectra of T90A-F3-c’; Figures S17–S21: Mass and NMR spectra of T90A-F3-d’; Figures S22–S24: The docking poses of chlorophyl1s to ZIKV proteins; Tables S1–S3: The chromatogram signal values of the purified compounds; Tables S4 and S5: The binding energies and the non-bond interaction between chlorophylls and ZIKV proteins

## References

[1] Liu W., Ruan R. (2022). Microalgae-based biomaterials for environmental remediation and functional use. Biomass, Biofuels, and Biochemicals.

[2] de Oliveira A.P.F., Bragotto A.P.A. (2022). Microalgae-based products: Food and public health. Future Foods.

[3] Martins T., Barros A.N., Rosa E., Antunes L. (2023). Enhancing health benefits through chlorophylls and chlorophyll-rich agro-food: A comprehensive review. Molecules.

[4] Spolaore P., Joannis-Cassan C., Duran E., Isambert A. (2006). Commercial applications of microalgae. J. Biosci. Bioeng..

[5] Balasubramaniam V., Gunasegavan R.D.-N., Mustar S., Lee J.C., Mohd Noh M.F. (2021). Isolation of industrial important bioactive compounds from microalgae. Molecules.

[6] Lanfer-Marquez U.M., Barros R.M.C., Sinnecker P. (2005). Antioxidant activity of chlorophylls and their derivatives. Food Res. Int..

[7] Ferruzzi M.G., Böhm V., Courtney P.D., Schwartz S.J. (2006). Antioxidant and antimutagenic activity of dietary chlorophyll derivatives determined by radical scavenging and bacterial reverse mutagenesis assays. J. Food Sci..

[8] Coulombier N., Jauffrais T., Lebouvier N. (2021). Antioxidant compounds from microalgae: A review. Mar. Drugs.

[9] Subramoniam A., Asha V.V., Nair S.A., Sasidharan S.P., Sureshkumar P.K., Rajendran K.N., Karunagaran D., Ramalingam K. (2012). Chlorophyll revisited: Anti-inflammatory activities of chlorophyll a and inhibition of expression of TNF-α gene by the same. Inflammation.

[10] Carvalho A.M.S., Heimfarth L., Pereira E.W.M., Oliveira F.S., Menezes I.R.A., Coutinho H.D.M., Picot L., Antoniolli A.R., Quintans J.S.S., Quintans-Júnior L.J. (2020). Phytol, a chlorophyll component, produces antihyperalgesic, anti-inflammatory, and antiarthritic effects: Possible NFκB pathway involvement and reduced levels of the proinflammatory cytokines TNF-α and IL-6. J. Nat. Prod..

[11] Ferruzzi M.G., Blakeslee J. (2007). Digestion, absorption, and cancer preventative activity of dietary chlorophyll derivatives. Nutr. Res..

[12] El-Sayed W.M., Hussin W.A., Mahmoud A.A., AlFredan M.A. (2013). The Conyza triloba extracts with high chlorophyll content and free radical scavenging activity had anticancer activity in cell lines. Biomed Res. Int..

[13] Li Y., Cui Y., Hu X., Liao X., Zhang Y. (2019). Chlorophyll supplementation in early life prevents diet-induced obesity and modulates gut microbiota in mice. Food Funct..

[14] da Silva Vaz B., Moreira J.B., de Morais M.G., Costa J.A.V.C. (2016). Microalgae as a new source of bioactive compounds in food supplements. Curr. Opin. Food Sci..

[15] Lauritano C., Helland K., Riccio G., Andersen J.H., Ianora A., Hansen E.H. (2020). Lysophosphatidylcholines and chlorophyll-derived molecules from the diatom Cylindrotheca Closterium with anti-inflammatory activity. Mar. Drugs.

[16] Lafarga T. (2020). Cultured microalgae and compounds derived thereof for food applications: Strain selection and cultivation, drying, and processing strategies. Food Rev. Int..

[17] Yadav G., Sen R. (2018). Sustainability of Microalgal Biorefinery: Scope, Challenges, and Opportunities. Sustainable Energy Technology and Policies.

[18] Zittelli G.C., Rodolfi L., Bassi N., Biondi N., Tredici M.R. (2013). Photobioreactors for Microalgal Biofuel Production. Algae for Biofuels and Energy.

[19] Cui X., Yang J., Cui M., Zhang W., Zhao J. (2021). Comparative experiments of two novel tubular photobioreactors with an inner aerated tube for microalgal cultivation: Enhanced mass transfer and improved biomass yield. Algal Res..

[20] Dammak M., Hadrich B., Miladi R., Barkallah M., Hentati F., Hachicha R., Laroche C., Michaud P., Fendri I., Abdelkafi S. (2017). Effects of nutritional conditions on growth and biochemical composition of *Tetraselmis* sp.. Lipids Health Dis..

[21] Bondioli P., Della Bella L., Rivolta G., Zittelli G.C., Bassi N., Rodolfi L., Casini D., Prussi M., Chiaramonti D., Tredici M.R. (2012). Oil production by the marine microalgae *Nannochloropsis* sp. F&M-M24 and *Tetraselmis suecica* F&M-M33. Bioresour. Technol..

[22] Schüler L.M., Bombo G., Duarte P., Santos T.F., Maia I.B., Pinheiro F., Marques J., Jacinto R., Schulze P.S.C., Pereira H. (2021). Carotenoid biosynthetic gene expression, pigment and n-3 fatty acid contents in carotenoid-rich *Tetraselmis striata* CTP4 strains under heat stress combined with high light. Bioresour. Technol..

[23] Kim T., Choi W.-S., Ye B.-R., Heo S.-J., Oh D., Kim S., Choi K.-S., Kang D.-H.J. (2018). Cultivating spirulina maxima: Innovative approaches. Cyanobacteria.

[24] Lee W.-K., Ryu Y.-K., Choi W.-Y., Kim T., Park A., Lee Y.-J., Jeong Y., Lee C.-G., Kang D.-H. (2021). Year-round cultivation of *Tetraselmis* sp. for essential lipid production in a semi-open raceway system. Mar. Drugs.

[25] Kim E.-A., Kang N., Heo S.-Y., Oh J.-Y., Lee S.-H., Cha S.-H., Kim W.-K., Heo S.-J. (2023). Antioxidant, antiviral, and anti-inflammatory activities of lutein-enriched extract of *Tetraselmis* species. Mar. Drugs.

[26] Pielnaa P., Al-Saadawe M., Saro A., Dama M.F., Zhou M., Huang Y., Huang J., Xia Z. (2020). Zika virus-spread, epidemiology, genome, transmission cycle, clinical manifestation, associated challenges, vaccine and antiviral drug development. Virology.

[27] de Sales-Neto J.M., Carvalho D.C.M., Magalhaes D.W.A., Medeiros A.B.A., Soares M.M., Rodrigues-Mascarenhas S. (2024). Zika virus: Antiviral immune response, inflammation, and cardiotonic steroids as antiviral agents. Int. Immunopharmacol..

[28] Marban-Castro E., Gonce A., Fumado V., Pomero-Acevedo L., Bardaji A. (2021). Zika virus infection in pregnant women and their children: A review. Eur. J. Obstet. Gynecol. Reprod. Biol..

[29] Liu Z.Y., Shi W.F., Qin C.F. (2019). The evolution of Zika virus from Asia to the Americas. Nat. Rev. Microbiol..

[30] Chen H., Lao Z., Xu J., Li Z., Long H., Li D., Lin L., Liu X., Yu L., Liu W. (2020). Antiviral activity of lycorine against Zika virus in vivo and in vitro. Viorology.

[31] Valcareggi Morcelli A., da Silva Andrade W., Frankenberg C.L.C., Rech R., Marcílio N.R. (2021). Extraction of chlorophylls and carotenoids from microalgae: Cosmo-sac-assisted solvent screening. Chem. Eng. Technol..

[32] Wood N.J., Baker A., Quinnell R.J., Camargo-Valero M.A. (2020). A simple and non-destructive method for chlorophyll quantification of Chlamydomonas cultures using digital image analysis. Front. Bioeng. Biotechnol..

[33] Ngcobo S., Bada S.O., Ukpong A.M., Risenga I. (2024). Optimal chlorophyll extraction conditions and postharvest stability in Moringa (*M. oleifera*) leaves. J. Food Meas. Charact..

[34] Tiji S., Rokni Y., Benayad O., Laaraj N., Asehraou A., Mimouni M. (2021). Chemical Composition related to antimicrobial activity of Moroccan Nigella sativa L. extracts and isolated fractions. Evid.-Based Complement. Altern. Med..

[35] Thabti I., Albert Q., Philippot S., Dupire F., Westerhuis B., Fontanay S., Risler A., Kassab T., Elfalleh W., Aferchichi A. (2020). Advances on antiviral activity of Morus spp. plant extracts: Human coronavirus and virus-related respiratory tract infections in the spotlight. Molecules.

[36] Reis A.C.C., Silva B.M., de Moura H.M.M., Pereira G.R., Brandão G.C. (2020). Anti-Zika virus activity and chemical characterization by ultrahigh performance liquid chromatography (UPLC-DAD-UV-MS) of ethanol extracts in Tecoma species. BMC Complement. Med. Ther..

[37] Sobrinho A.C.N., de Morais S.M., Marinho M.M., de Souza N.V., Lima D.M. (2021). Antiviral activity on the Zika virus and larvicidal activity on the *Aedes* spp. of *Lippia alba* essential oil and β-caryophyllene. Ind. Crops. Prod..

[38] Katze M.G., He Y., Gale M.G. (2002). Viruses and interferon: A fight for supremacy. Nat. Rev. Immunol..

[39] Diamond M.S., Farzan M. (2013). The broad-spectrum antiviral functions of IFIT and IFITM proteins. Nat. Rev. Immunol..

[40] Kang S., Brown H.M., Hwang S. (2018). Direct Antiviral Mechanisms of Interferon-Gamma. Immune Netw..

[41] Cordeiro P.A.S., Assone T., Prates G., Tedeschi M.R.M., Fonseca L.A.M., Casseb J. (2022). The role of IFN-γ production during retroviral infections: An important cytokine involved in chronic inflammation and pathogenesis. Rev. Inst. Med. Trop. Sao. Paulo..

[42] Couto J.C.M., Vidal T., Decker E.R., Santurio J.M., Mello C.F., Pillat M.M. (2023). Use of recombinant S1 protein with hFc for analysis of SARS-CoV-2 adsorption and evaluation of drugs that inhibit entry into VERO E6 cells. Immunol. Lett..

[43] Lee J.K., Nam H.G., Zare R.N. (2017). Microdroplet fusion mass spectrometry: Accelerated kinetics of acid-induced chlorophyll demetallation. Q. Rev. Biophys..

[44] Gliszczyńska A., Dancewicz K., Gabryś B., Świtalska M., Wietrzyk J., Maciejewska G. (2021). Synthesis of novel phytol-derived γ-butyrolactones and evaluation of their biological activity. Sci. Rep..

[45] Jimenez-Aleman G.H., Castro V., Londaitsbehere A., Gutierrez-Rodríguez M., Garaigorta U., Solano R., Gastaminza P. (2021). SARS-CoV-2 fears green: The chlorophyll catabolite pheophorbide a is a potent antiviral. Pharmaceuticals.

[46] Meunier T., Desmarets L., Bordage S., Bamba M., Hervouet K., Rouillé Y., François N., Decossas M., Sencio V., Trottein F. (2022). A photoactivable natural product with broad antiviral activity against enveloped viruses, including highly pathogenic coronaviruses. Antimicrob. Agents Chemother..

[47] Ratnoglik S.L., Aoki C., Sudarmono P., Komoto M., Deng L., Shoji I., Fuchino H., Kawahara N., Hotta H. (2014). Antiviral activity of extracts from Morinda citrifolia leaves and chlorophyll catabolites, pheophorbide a and pyropheophorbide a, against hepatitis C virus. J. Microbiol. Immunol. Infect..

[48] Kamei Y., Aoki M. (2007). A chlorophyll c2 analogue from the marine brown alga Eisenia bicyclis inactivates the infectious hematopoietic necrosis virus, a fish rhabdovirus. Arch. Virol..

[49] Gorshkov K., Shiryaev S.A., Fertel S., Lin Y.W., Huang C.T., Pinto A., Farhy C., Strongin A.Y., Zheng W., Tershikh A.V. (2018). Zika Virus: Origins, Pathological Action, and Treatment Strategies. Front. Microbiol..

[50] Fernandes A.S., Nascimento T.C., Pinheiro P.N., de Rosso V.V., de Menezes C.R., Jacob-Lopes E., Zepka L.Q. (2021). Insights on the intestinal absorption of chlorophyll series from microalgae. Food Res. Int..

[51] Kang N., Kim E.-A., Park A., Heo S.-Y., Heo J.-H., Heo S.-J. (2024). Antiviral potential of fucoxanthin, an edible carotenoid purified from *Sargassum siliquastrum*, against Zika virus. Mar. Drugs.

[52] Chomczynski P., Sacchi N. (2006). The single-step method of RNA isolation by acid guanidinium thiocyanate–phenol–chloroform extraction: Twenty-something years on. Nat. Protoc..

[53] Lim T., Rajoriya S., Kim B., Natasha A., Im H., Shim H.S., Yoo J., Kim J.W., Lee E.-W., Shin H.J. (2024). In vitro broad-spectrum antiviral activity of MIT-001, a mitochondria-targeted reactive oxygen species scavenger, against severe acute respiratory syndrome coronavirus 2 and multiple zoonotic viruses. Virus Res..

[54] Aoki-Utsubo C., Chen M., Hotta H. (2018). Virucidal and Neutralizing Activity Tests for Antiviral Substances and Antibodies. Bio Protoc..

[55] Kang N., Heo S.-Y., Kim E.-A., Cha S.-H., Ryu B., Heo S.-J. (2023). Antiviral effect of fucoxanthin obtained from *Sargassum siliquastrum* (Fucales, Phaeophyceae) against severe acute respiratory syndrome coronavirus 2. Algae.

[56] Kang N., Kim E.-A., Heo S.-Y., Heo S.-J. (2023). Structure-based in silico screening of marine phlorotannins for potential walrus calicivirus inhibitor. Int. J. Mol. Sci..

