# Supplementary material for: Antiviral Activity of Chlorophyll Extracts from Tetraselmis sp., a Marine Microalga, Against Zika Virus Infection
Source: Mar Drugs. 2024 Aug 31;22(9):397. doi: 10.3390/md22090397 (PMC11433109; doi:10.3390/md22090397)
Supplement: Supplementary file 1 [file marinedrugs-22-00397-s001.zip › marinedrugs-3139205-supplementary.pdf]

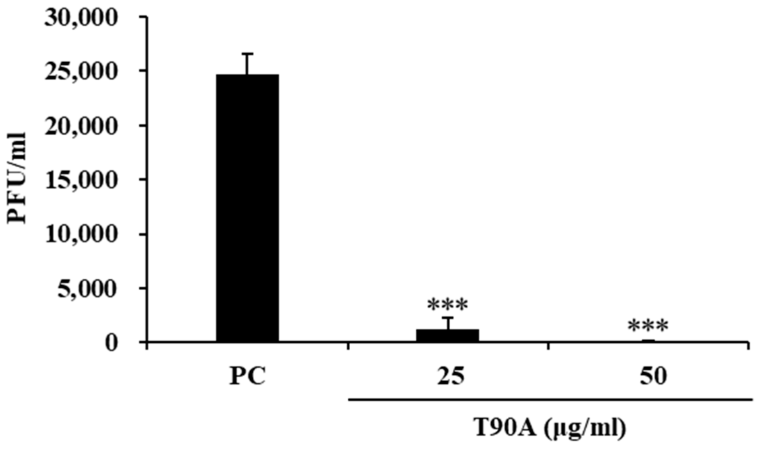

Figure S1. Inhibition effects of T90A on ZIKV infection

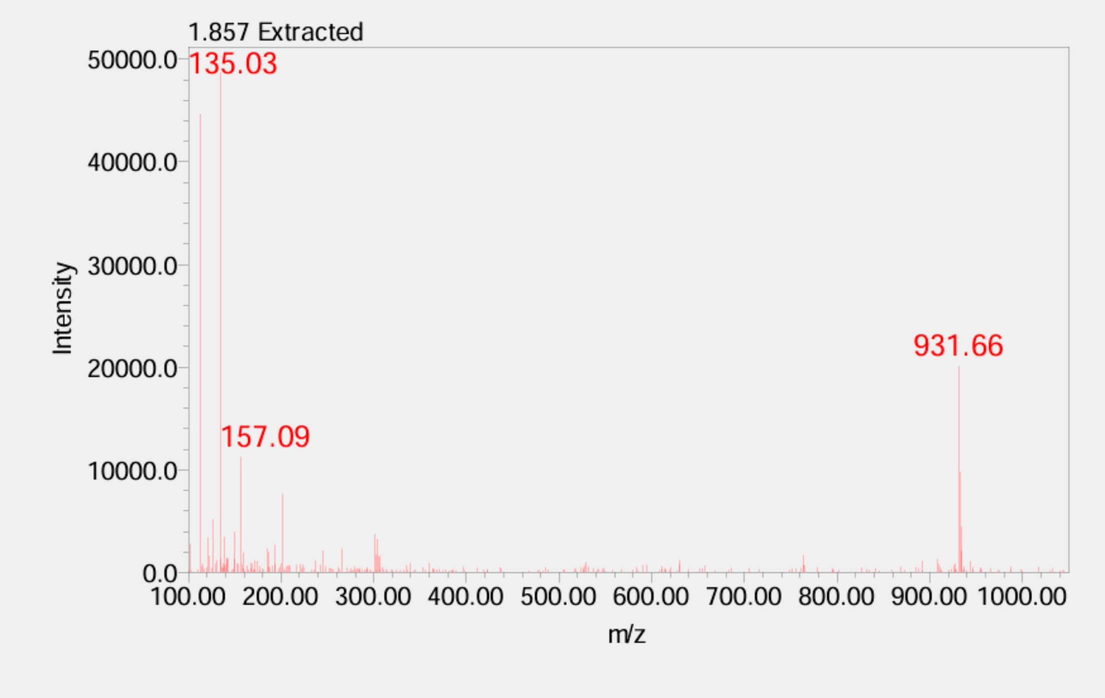

Figure S2. Mass spectrum of T90A-F3-a’

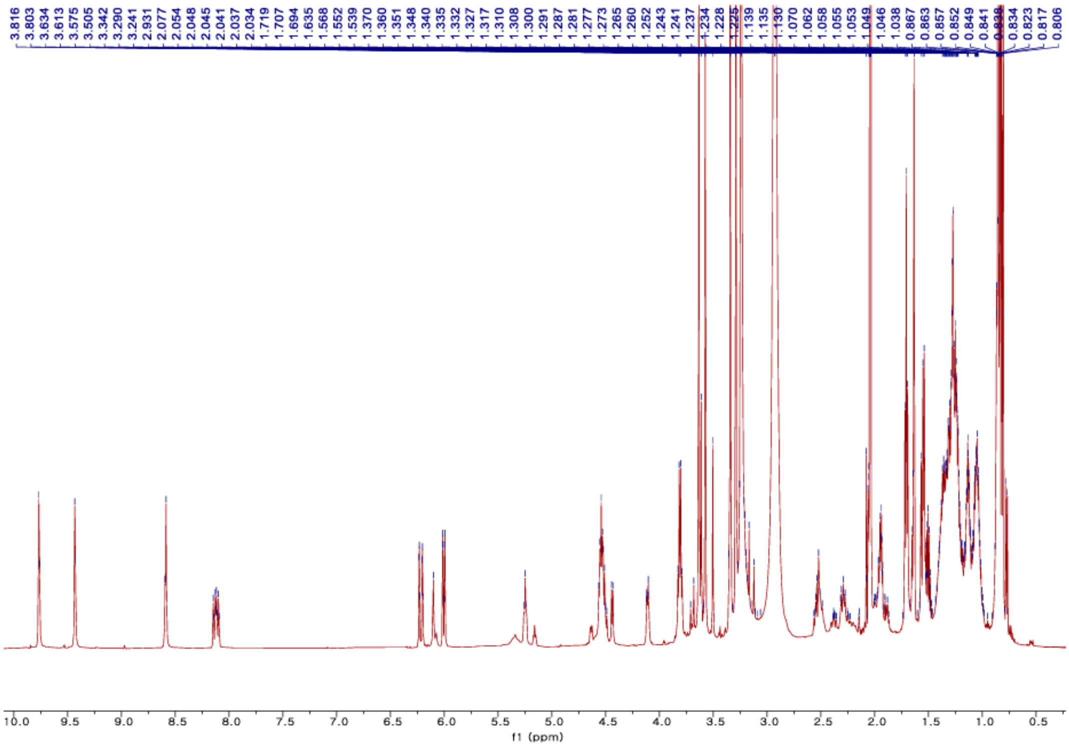

Figure S3. 1H-NMR spectrum of T90A-F3-a’

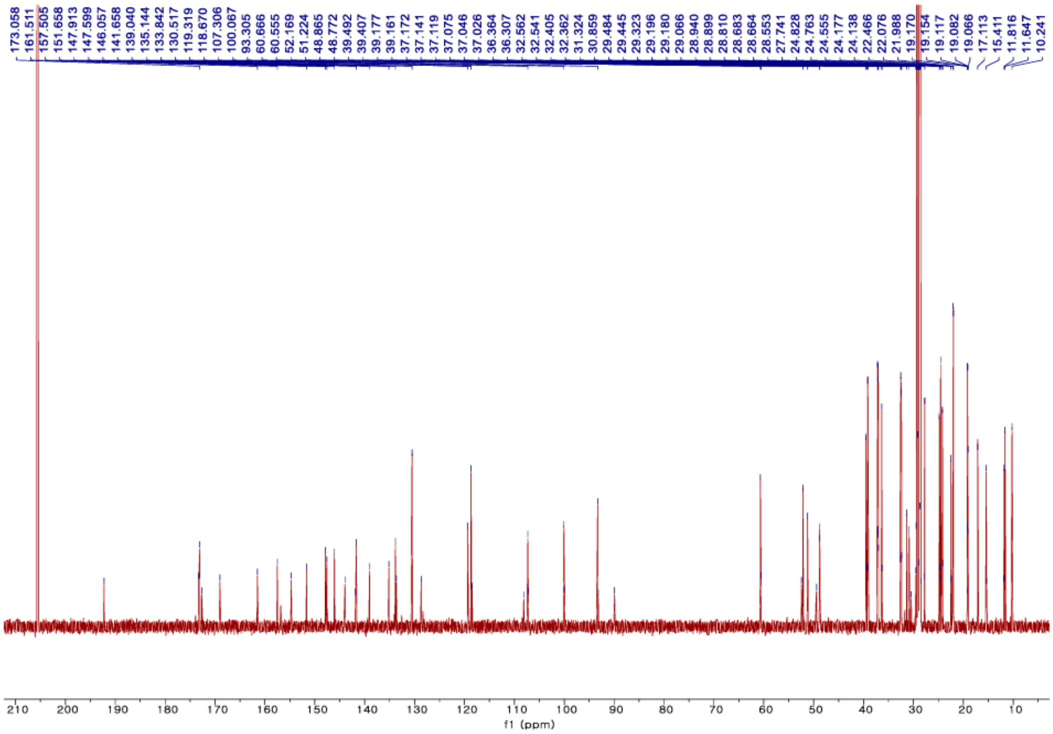

Figure S4. 13C-NMR spectrum of T90A-F3-a’

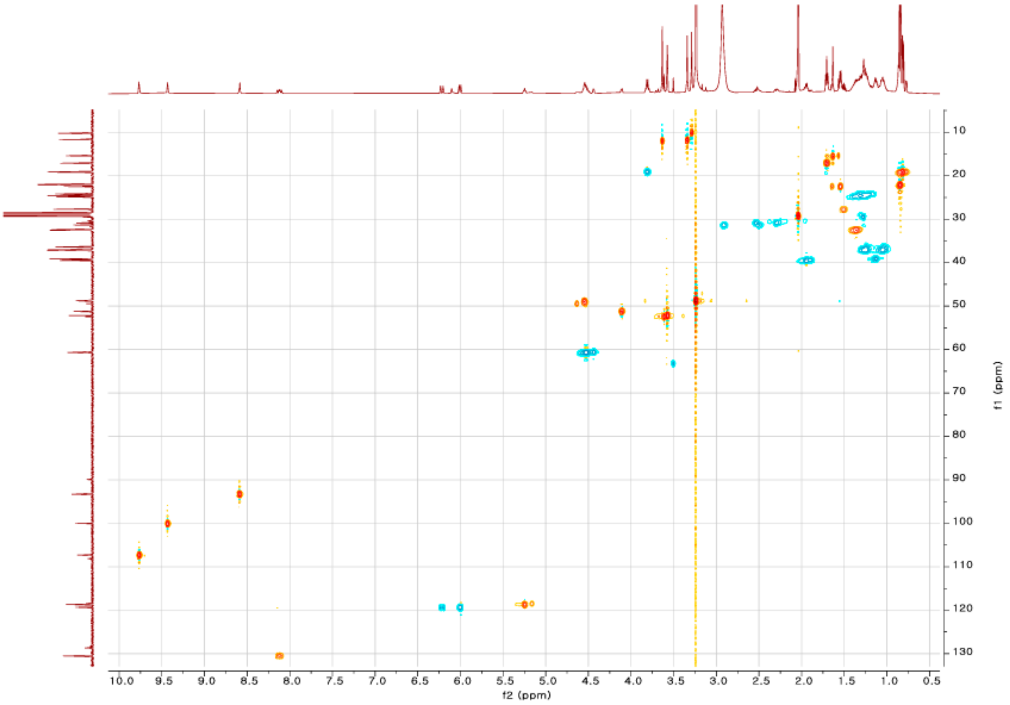

Figure S5. HSQC spectrum of T90A-F3-a’

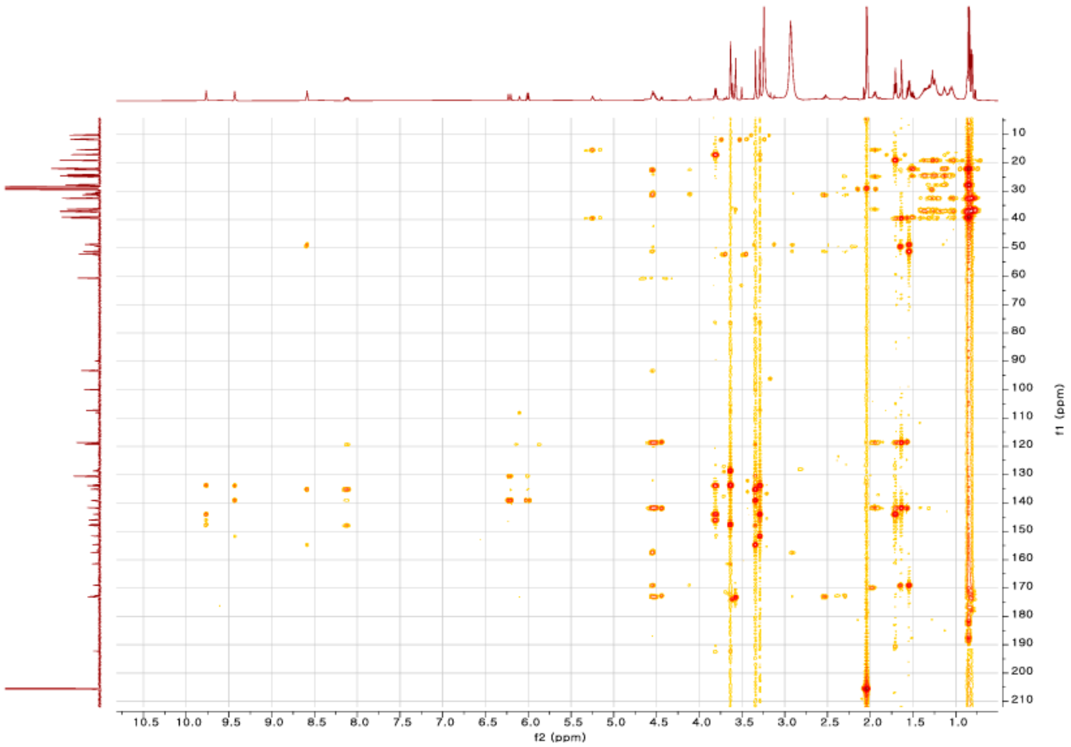

Figure S6. HMBC spectrum of T90A-F3-a’

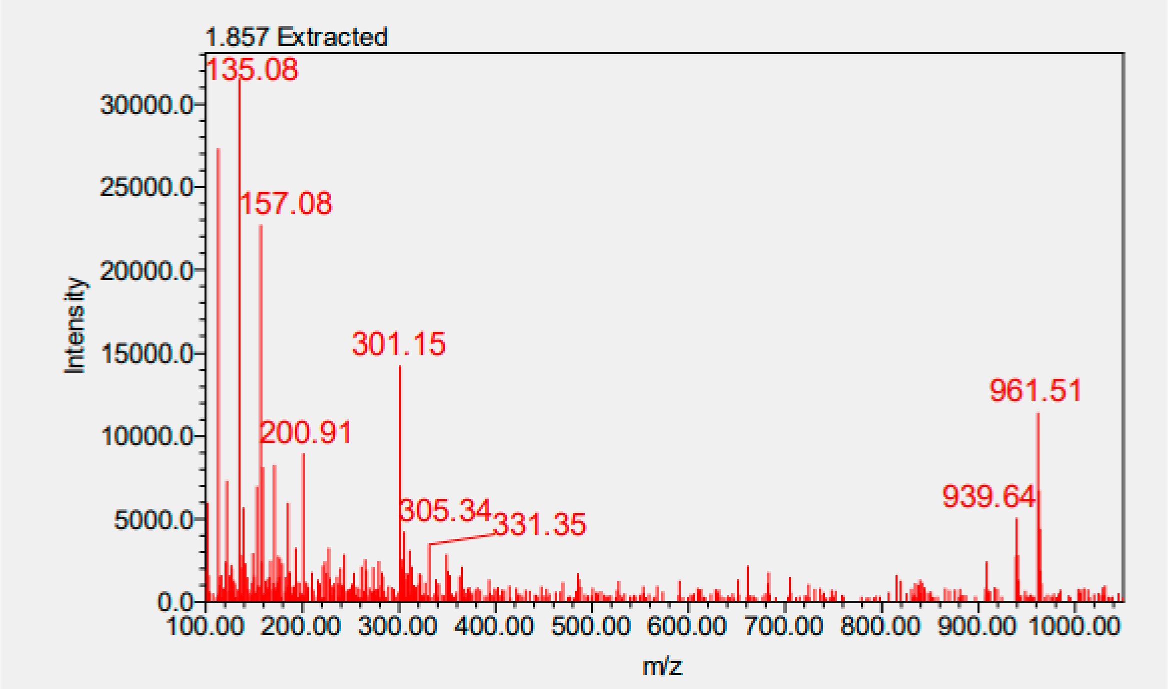

Figure S7. Mass spectrum of T90A-F3-b’

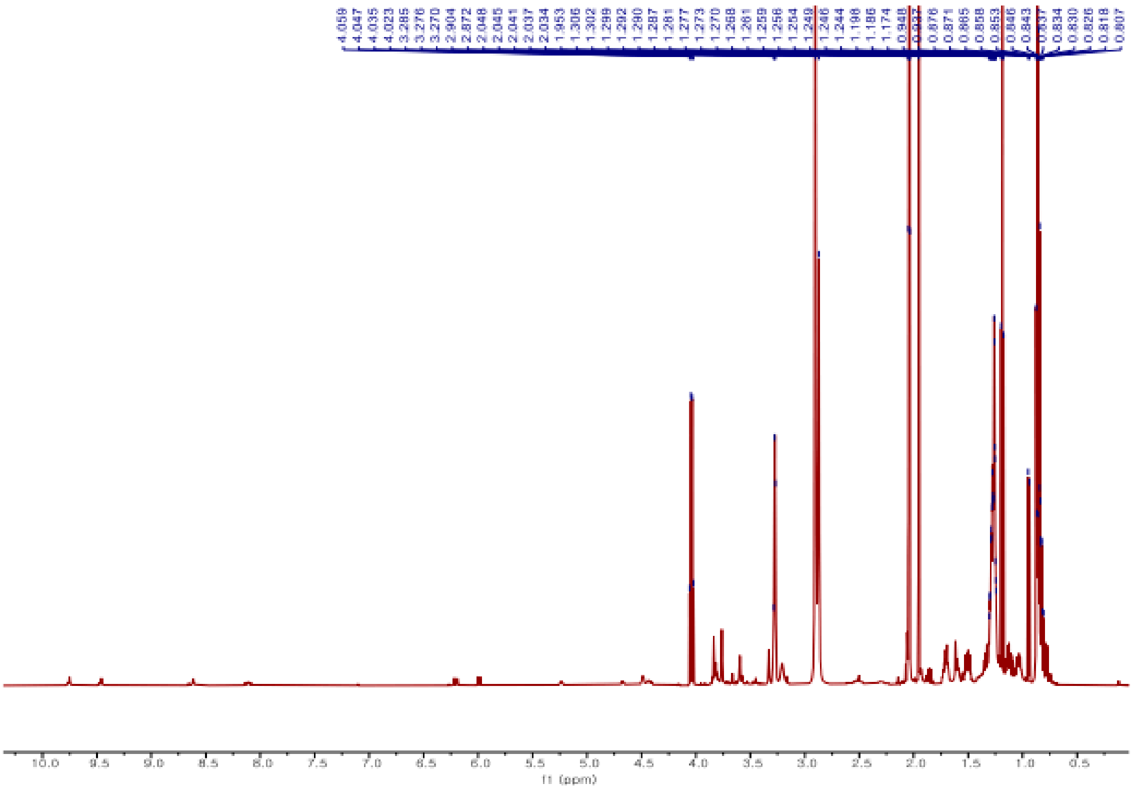

Figure S8. 1H-NMR spectrum of T90A-F3-b’

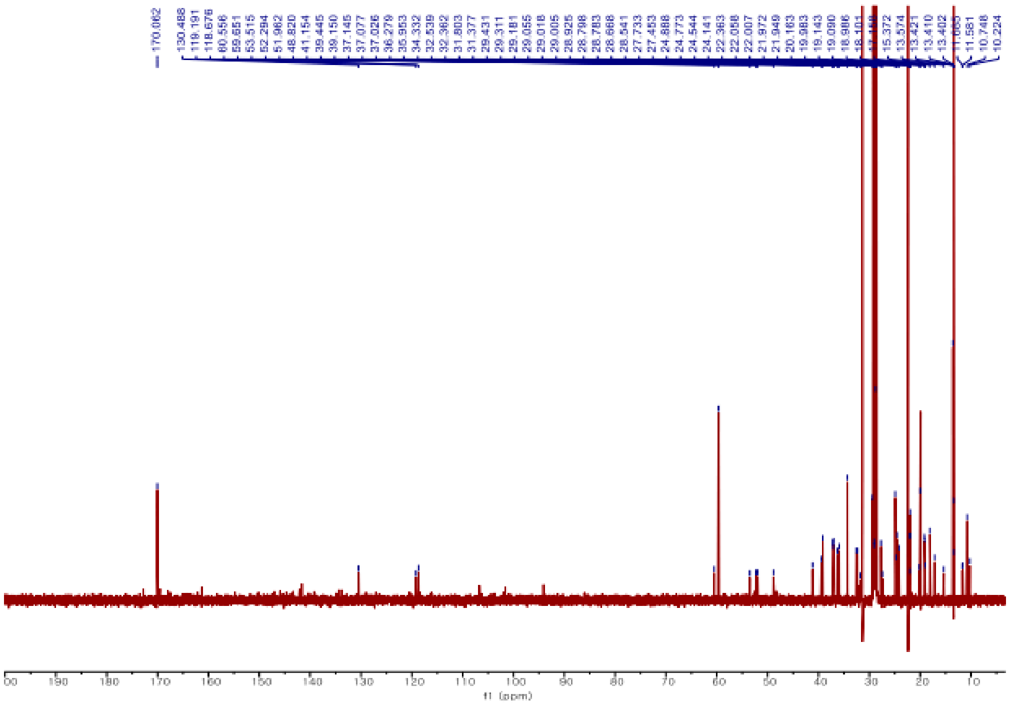

Figure S9. 13C-NMR spectrum of T90A-F3-b'

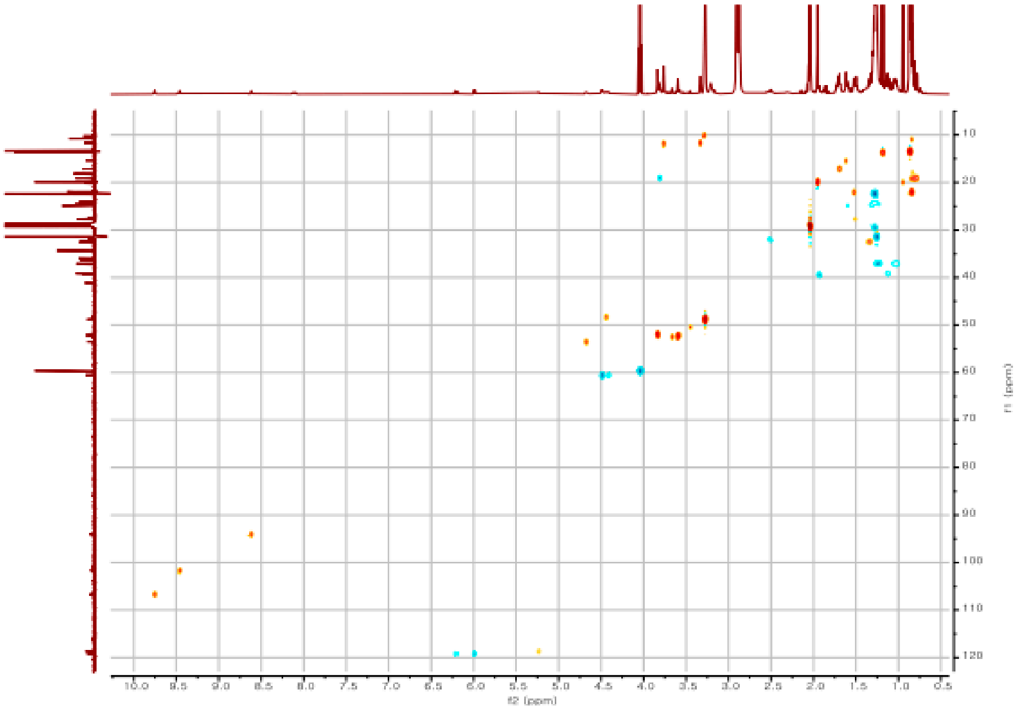

Figure S10. HSQC spectrum of T90A-F3-b'

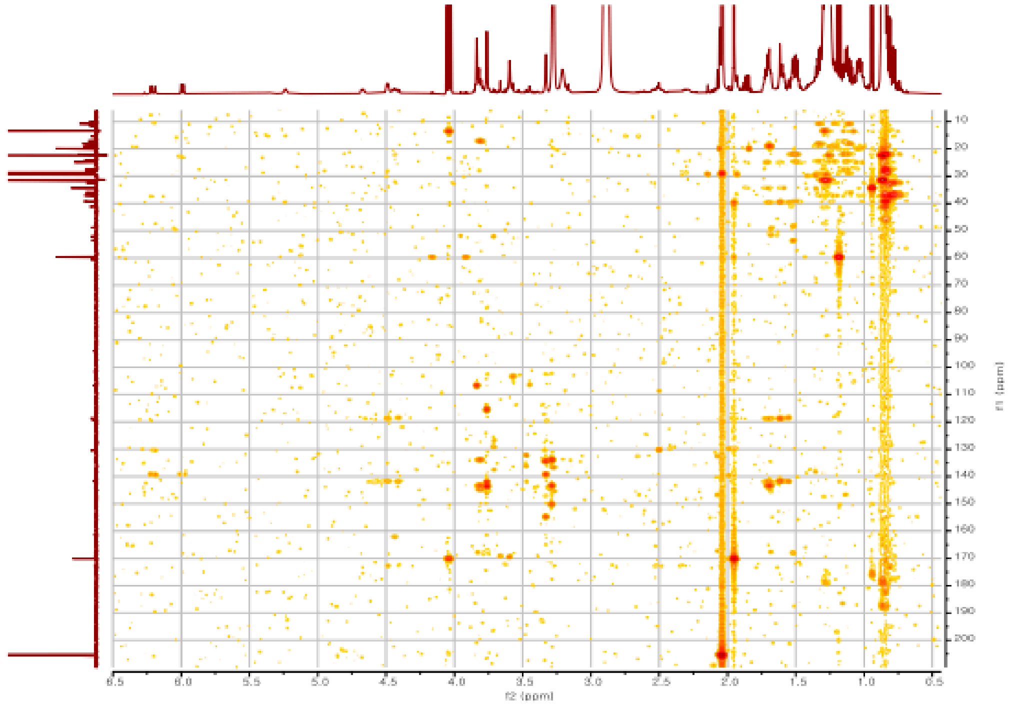

Figure S11. HMBC spectrum of T90A-F3-b’

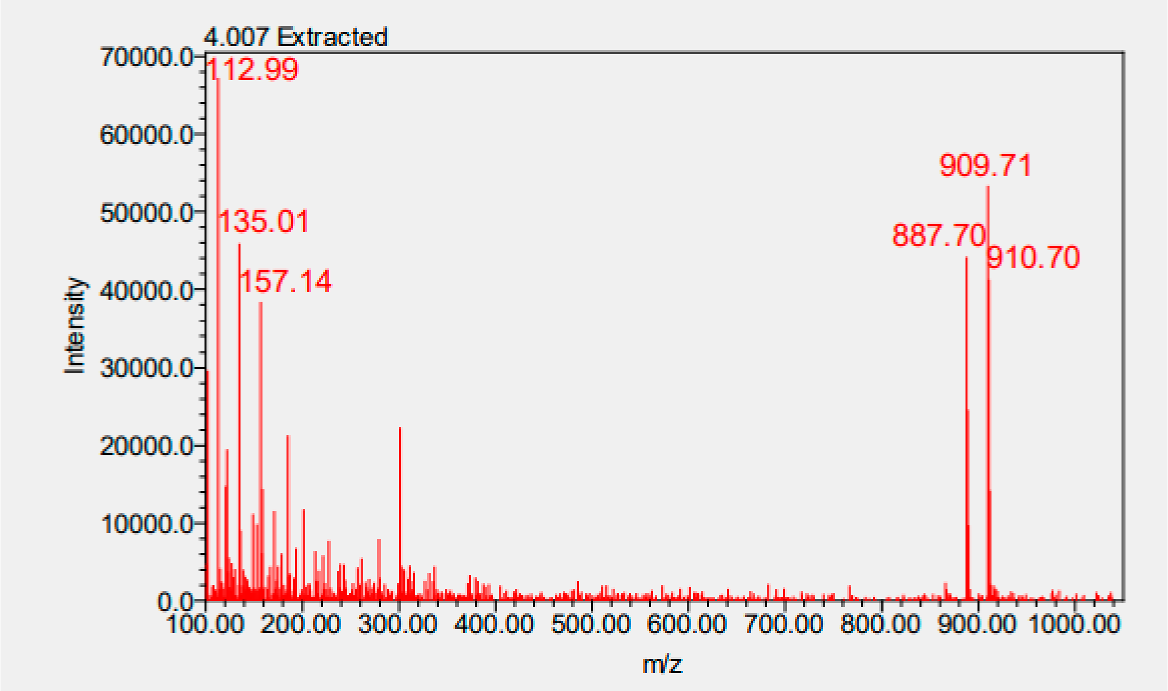

Figure S12. Mass spectrum of T90A-F3-c'

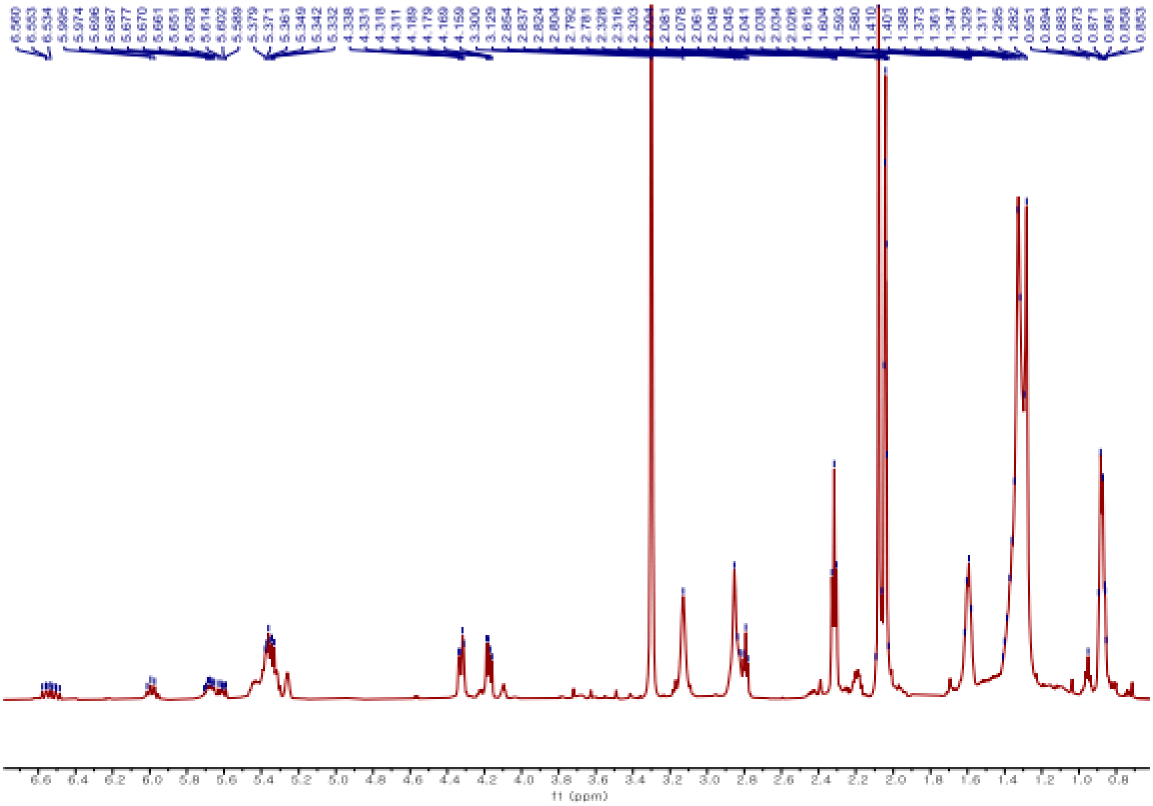

Figure S13. 1H-NMR spectrum of T90A-F3-c'

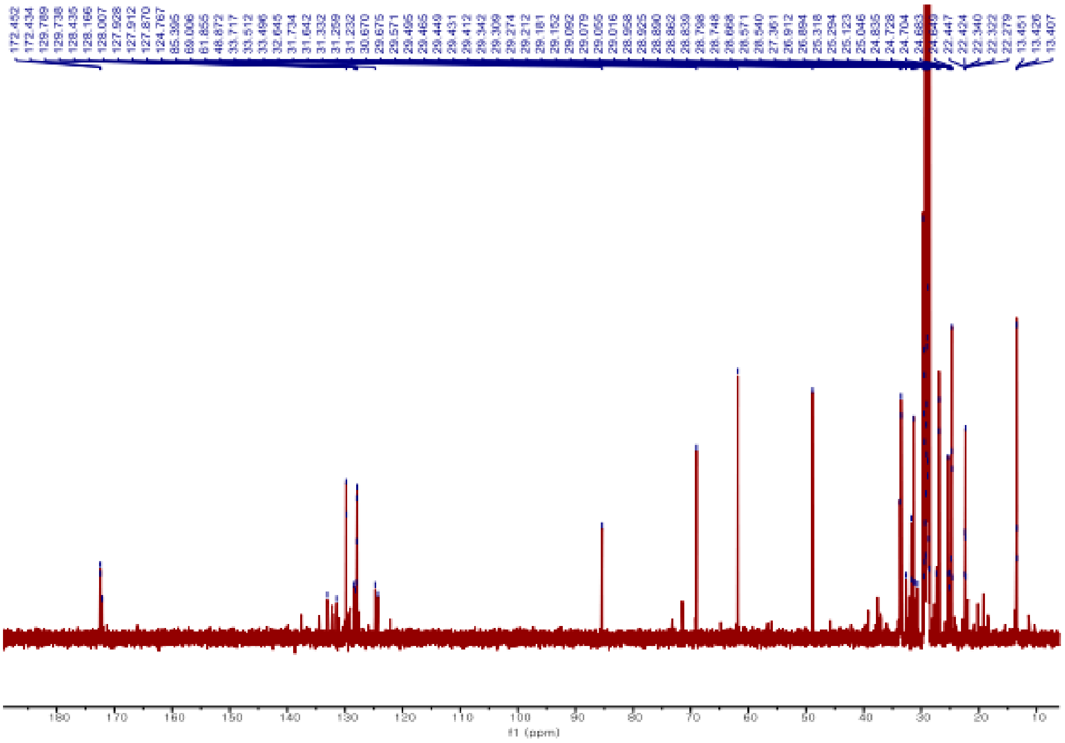

Figure S14. 13C-NMR spectrum of T90A-F3-c’

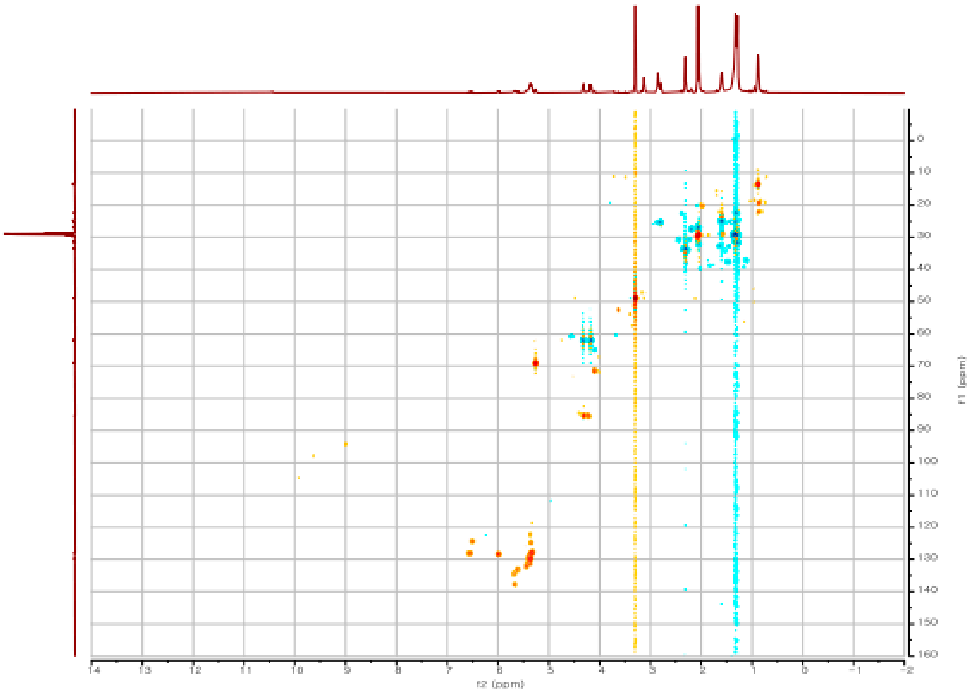

Figure S15. HSQC spectrum of T90A-F3-c’

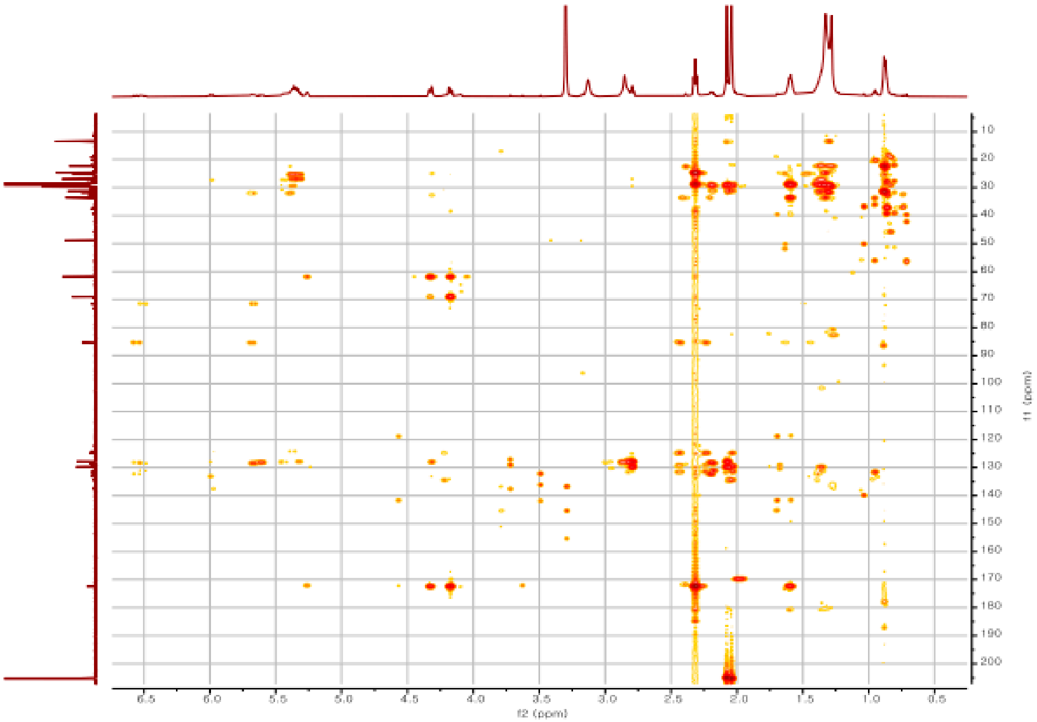

Figure S16. HMBC spectrum of T90A-F3-c’

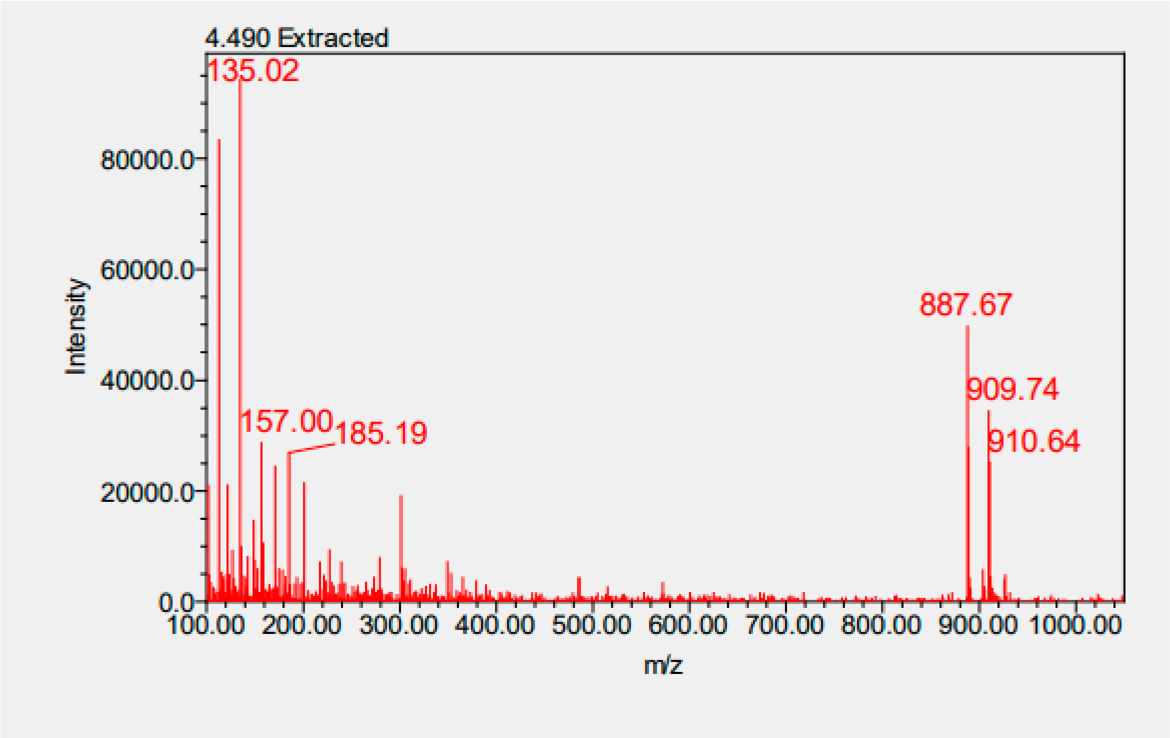

Figure S17. Mass spectrum of T90A-F3-d’

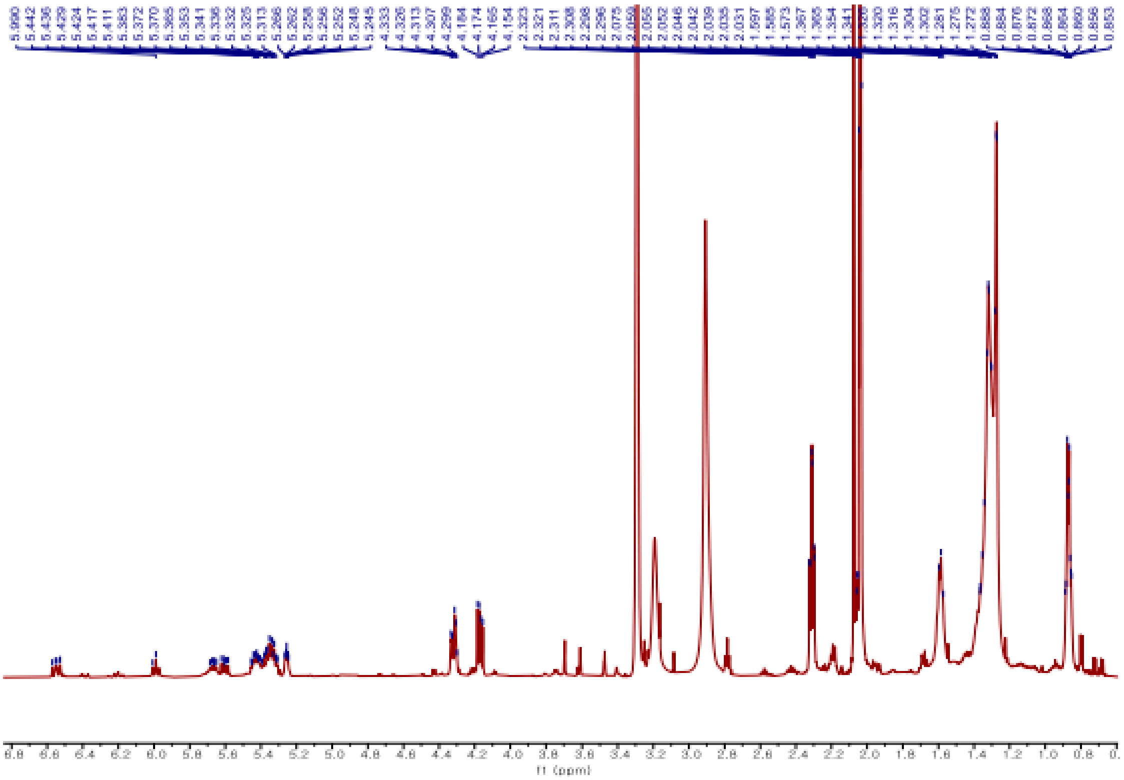

Figure S18. 1H-NMR spectrum of T90A-F3-d’

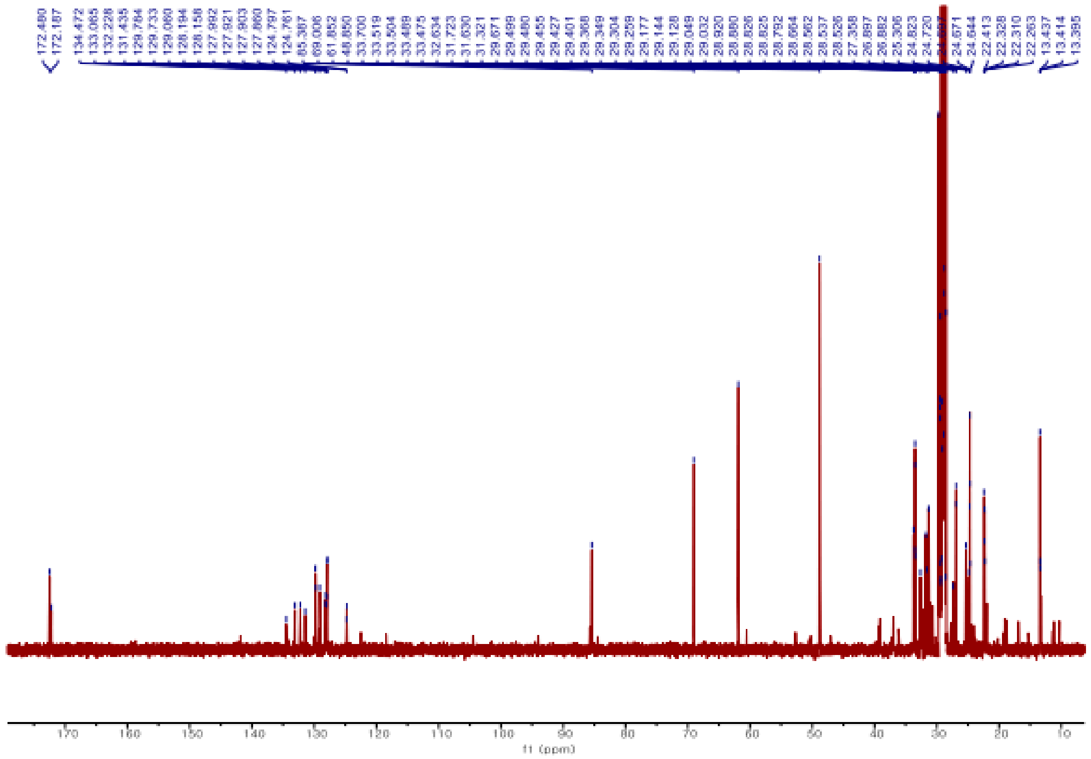

Figure S19. 13C-NMR spectrum of T90A-F3-d’

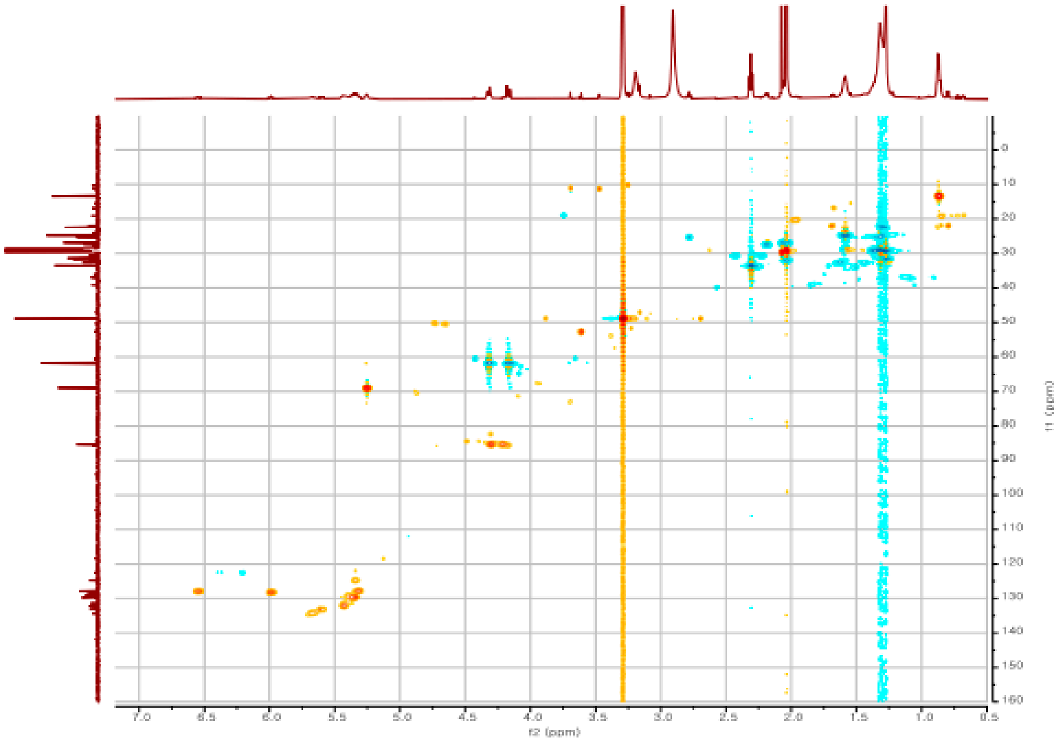

Figure S20. HSQC spectrum of T90A-F3-d’

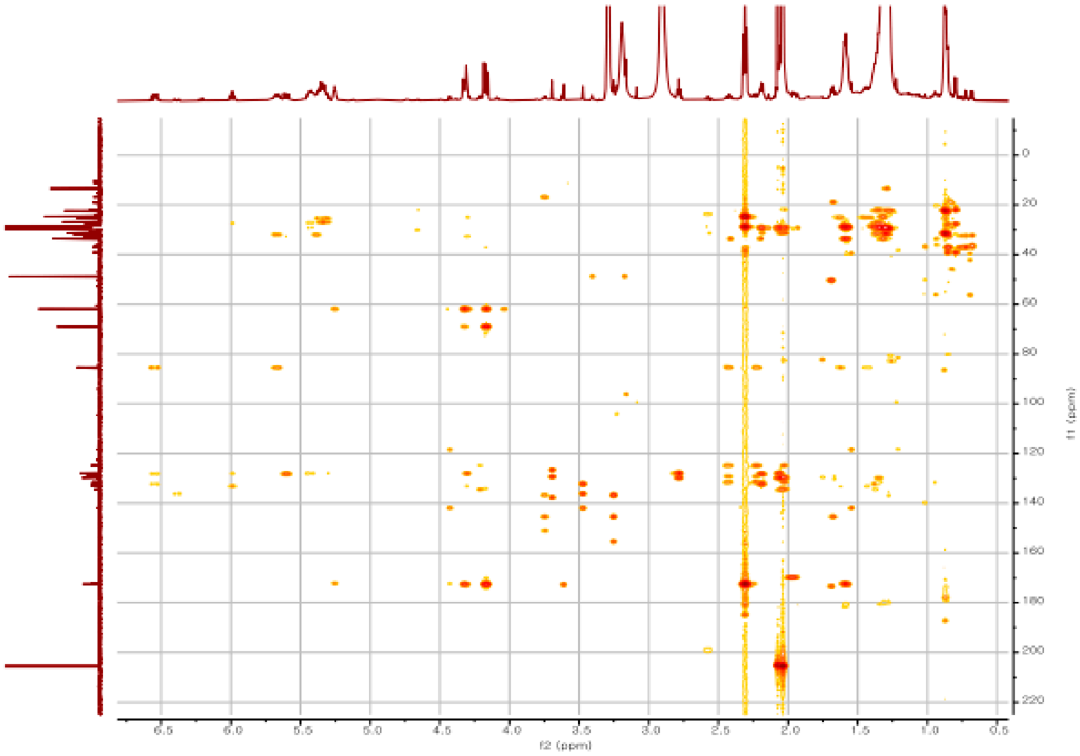

Figure S21. HMBC spectrum of T90A-F3-d’

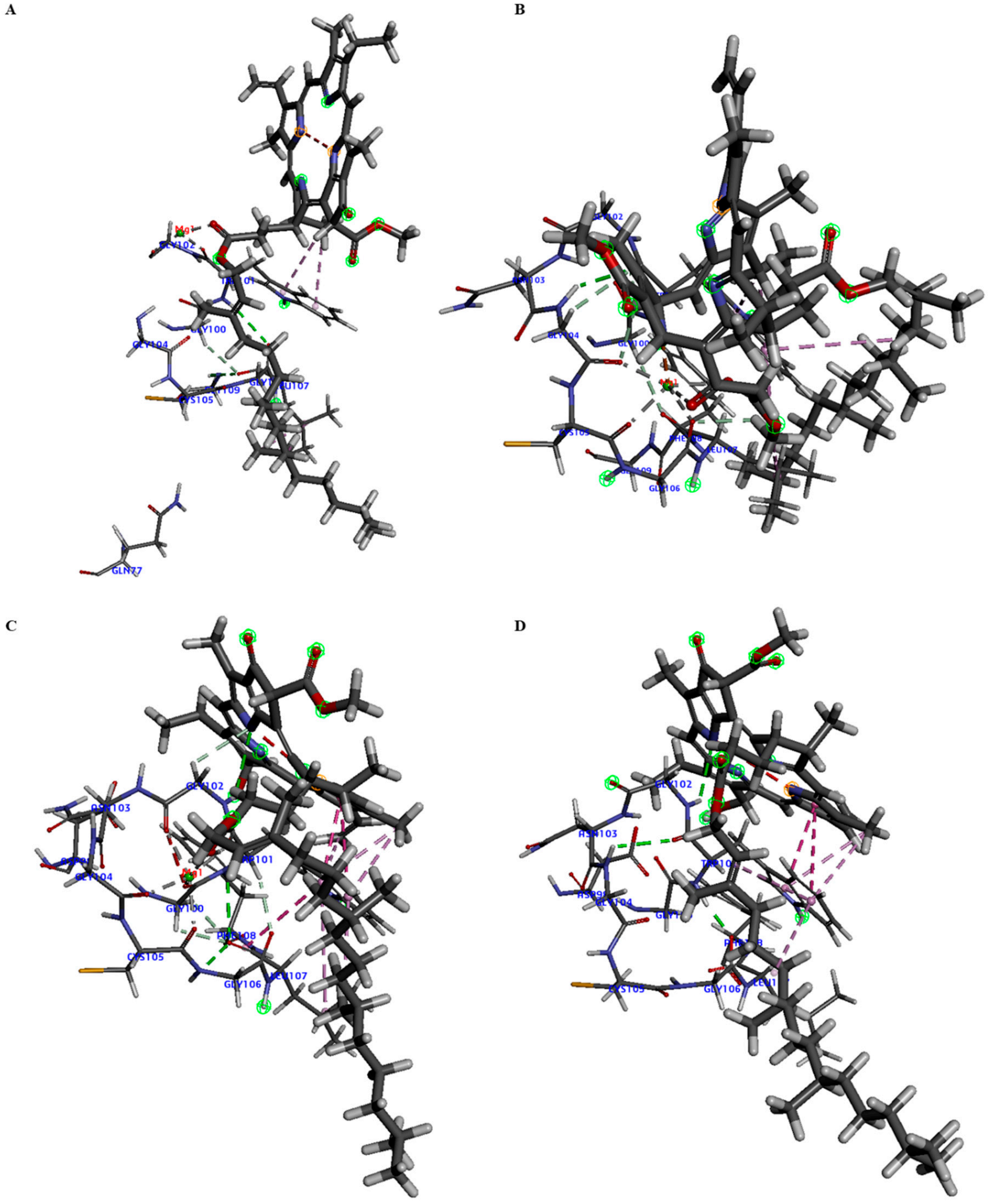

Figure S22. The docking poses of chlorophyl1s to zika virus envelop protein (5JHM). Chlorophyll A (A), chlorin e6-131-152-dimethyl-173-phytyl ester (B), hydroxychlorophyll A (C), and hydroxypheophytin A (D).

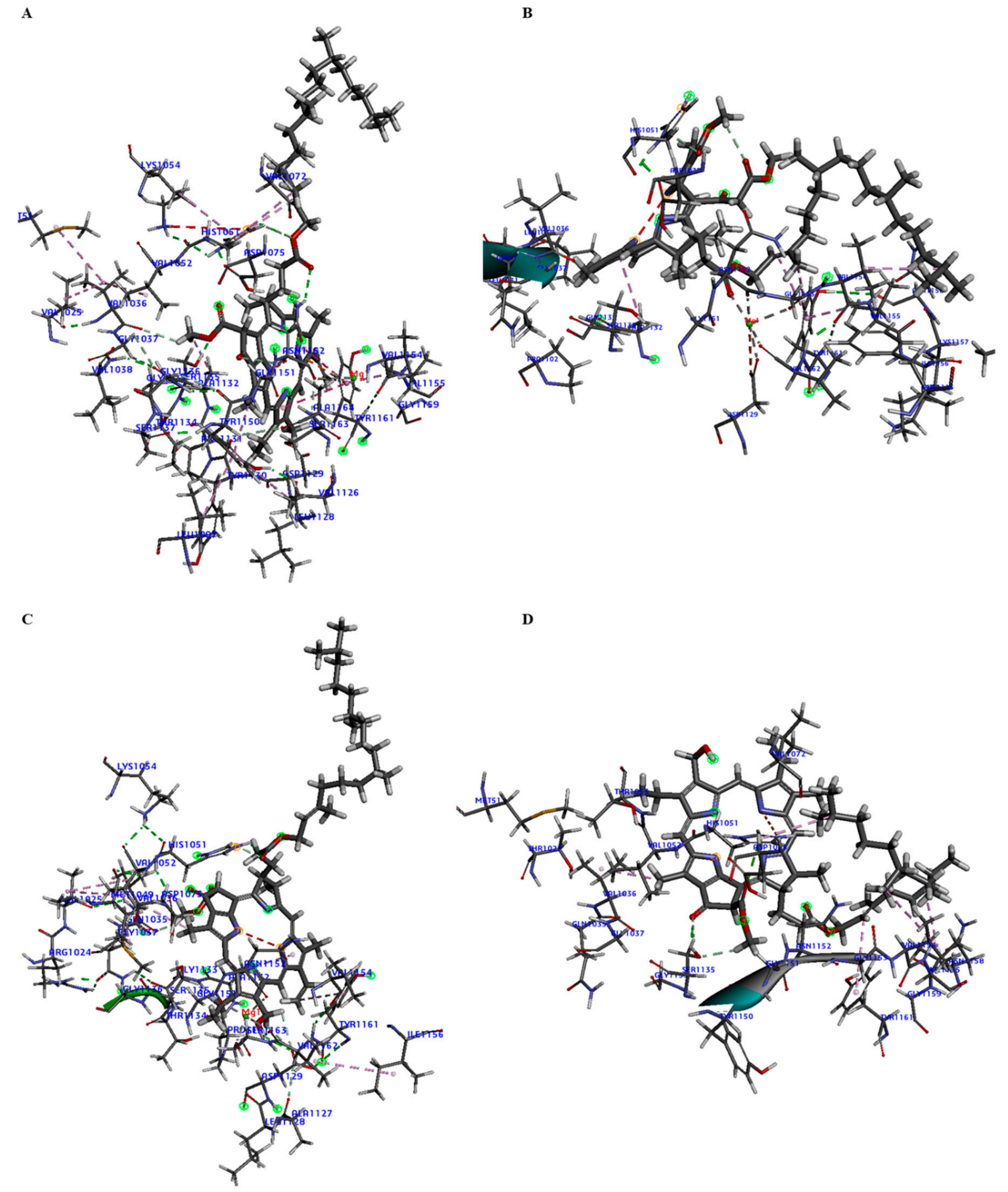

Figure S23. The docking poses of chlorophyl1s to zika virus NS2B/NS3 (5LC0). Chlorophyll A (A), chlorin e6-131-152-dimethyl-173-phytyl ester (B), hydroxychlorophyll A (C), and hydroxypheophytin A (D).

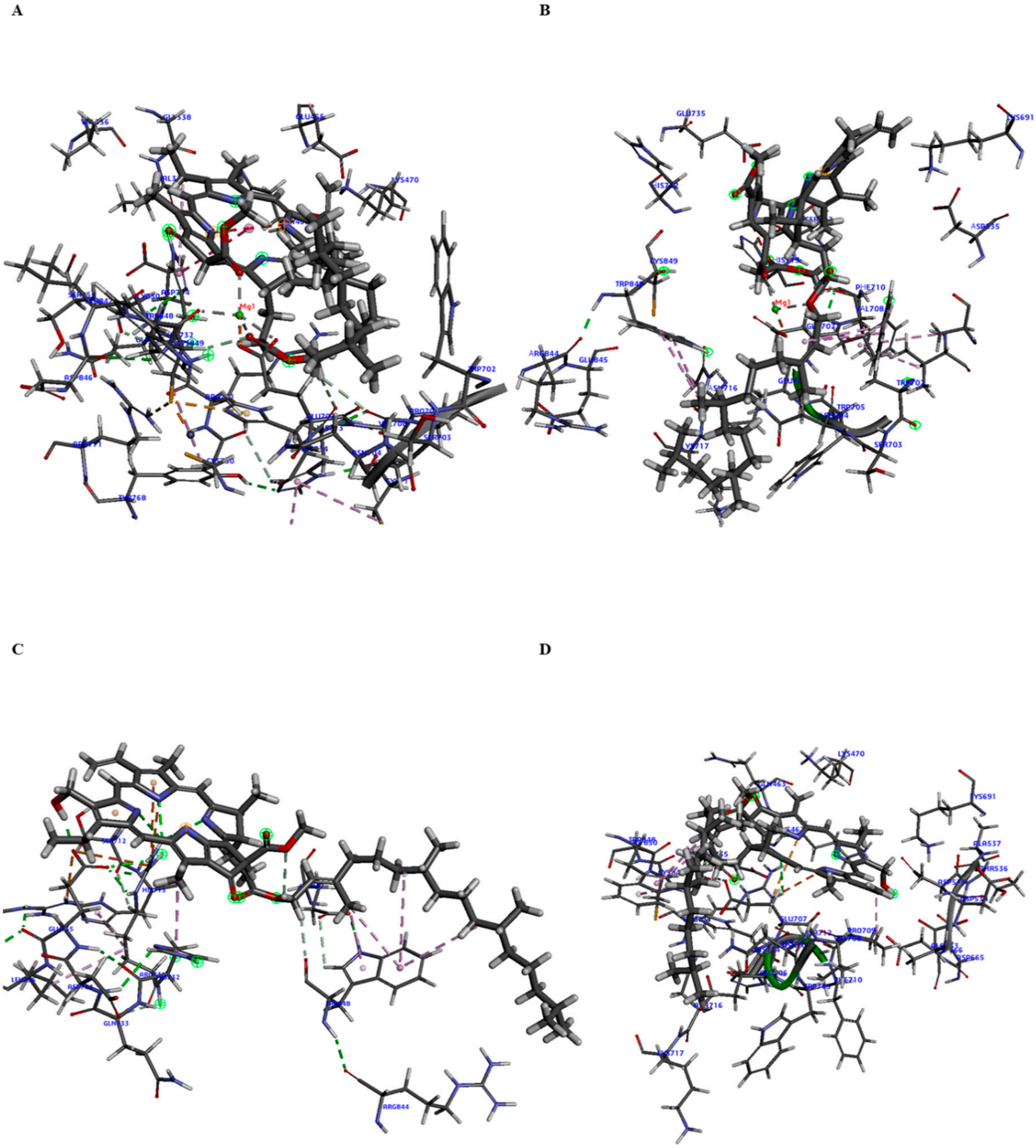

Figure S24. The docking poses of chlorophyl1s to zika virus RdRp (5TFR). Chlorophyll A (A), chlorin e6-131-152-dimethyl-173-phytyl ester (B), hydroxychlorophyll A (C), and hydroxypheophytin A (D).

Table S1. The chromatogram signal values of T90A-F3-a'.

No.	RT	Area	% Area	Height	Denotation	Purity
**1**	7.185	563,223	1.13	66,421		
**2**	7.509	2,710,245	5.43	79,377		
**3**	9.403	2,059,629	4.13	182,586		
**4**	9.742	4,970,304	9.97	201,985		
**5**	10.311	13,685,517	27.44	324,309		
**6**	14.374	1,847,745	3.70	39,916		
**7**	15.204	24,036,018	48.19	2,026,402	T90A-F3-a'	99.08%

Table S2. The chromatogram signal values of T90A-F3-b'.

No.	RT	Area	% Area	Height	Denotation	Purity
**1**	7.134	7,419	0.01	620		
**2**	7.390	17,752	0.03	3,602		
**3**	12.188	13,931	0.03	1,109		
**4**	13.078	44,326,154	80.99	2,009,474	T90A-F3-b'	98.05%
**5**	23.040	10,364,223	18.94	224,979		

Table S3. The chromatogram signal values of T90A-F3-c’ and T90A-F3-d’.

No.	RT	Area	% Area	Height	Denotation	Purity
**1**	6.267	105,061	0.23	1,426		
**2**	8.433	24,234	0.05	1,187		
**3**	8.883	93,888	0.21	1,896		
**4**	10.041	157,505	0.35	3,307		
**5**	29.233	228,021	0.50	6,582		
**6**	31.817	10,955,148	24.25	124,283	T90A-F3-c’	94.68%
**7**	34.914	27,951,798	61.88	307,649	T90A-F3-d’	99.61%

Table S4. The calculated binding energies between chlorophylls (hydroxychlorophyll a and hydroxypheophytin a) and zika virus proteins.

**Chlorophyll**	**Protein**	**Binding energy (kcal/mol)**
Hydroxychlorophyll A	Envelope protein	-511.311
	NS2B/NS3	-751.503
	RdRp	-899.910
Hydroxypheophytin A	Envelope protein	-122.571
	NS2B/NS3	-286.441
	RdRp	-337.940

Table S5. The non-bond interaction between chlorophylls chlorophylls (hydroxychlorophyll a and hydroxypheophytin a) and Zika virus proteins.

Chlorophyll	Protein	Non-bond interaction in active site
Hydroxychlorophyll A	Envelope protein	Mg^{2+}-GLY100, Mg^{2+}-TRP101, Mg^{2+}-GLY102, Mg^{2+}-GLY104, Mg^{2+}-CYS105, C3-TRP101 (6), C3-GLY102 (2), C3-LEU107
	NS2B/NS3	Mg^{2+}-GLY1151, Mg^{2+}-ASN1152, Mg^{2+}-VAL1162, Mg^{2+}-SER1163, C3-GLN1035, C3-VAL1036, C3-HIS1051 (2), C3-VAL1052, C3-ASP1129 (3), C3-ALA1132, C3-TYR1161, C3-VAL1162
	RdRp	C3-ARG731 (5), C3-HIS732, C3-GLU735 (2), C3-TRP848 (5)
Hydroxypheophytin A	Envelope protein	C4-TRP101 (5), C4-GLY102
	NS2B/NS3	C4-VAL1036, C4-HIS1051 (4), C4-SER1135 (3), C4-VAL1154, C4-VAL1155, C4-TYR1161
	RdRp	C4-GLU707, C4-PRO709, C4-ARG731 (3), C4-TRP848 (3), C4-CYS849

The number in parentheses indicates the number of the non-bond interactions.
